# Microbial ecology of subsurface granitic bedrock: a humid–arid site comparison in Chile

**DOI:** 10.1093/ismeco/ycaf199

**Published:** 2025-11-04

**Authors:** Lucas Horstmann, Daniel Lipus, Alexander Bartholomäus, Romulo Oses, Axel Kitte, Thomas Friedl, Dirk Wagner

**Affiliations:** GFZ Helmholtz Centre for Geosciences, Section Geomicrobiology, Telegrafenberg, Potsdam 14473, Germany; Department Experimental Phycology and Culture Collection of Algae (EPSAG), Albrecht-von-Haller-Institute for Plant Sciences, Georg August University Göttingen, Nikolausberger Weg 18, Göttingen 37073, Germany; GFZ Helmholtz Centre for Geosciences, Section Geomicrobiology, Telegrafenberg, Potsdam 14473, Germany; GFZ Helmholtz Centre for Geosciences, Section Geomicrobiology, Telegrafenberg, Potsdam 14473, Germany; Centro Regional de Investigación y Desarrollo Sustentable de Atacama (CRIDESAT), Universidad de Atacama, Copiapó 1530000, Chile; GFZ Helmholtz Centre for Geosciences, Section Geomicrobiology, Telegrafenberg, Potsdam 14473, Germany; Department Experimental Phycology and Culture Collection of Algae (EPSAG), Albrecht-von-Haller-Institute for Plant Sciences, Georg August University Göttingen, Nikolausberger Weg 18, Göttingen 37073, Germany; GFZ Helmholtz Centre for Geosciences, Section Geomicrobiology, Telegrafenberg, Potsdam 14473, Germany; Institute of Geosciences, University of Potsdam, Karl-Liebknecht-Str. 24–25, Potsdam 14476, Germany

**Keywords:** subsurface environments, desert, microbial communities, chemolithoautotrophy, microbial weathering, granite, terrestrial subsurface

## Abstract

Subsurface microorganisms face extreme challenges such as anoxic, xeric, and oligotrophic conditions. In igneous systems, nutrient limitation is critical, as biomass input relies on surface-derived fluids via tectonic fractures. Despite growing interest in subsurface habitats, little is known about ecosystems beneath arid landscapes, where surface water input is limited by the low annual precipitation. This study compares granitic subsurface environments beneath arid and humid surface ecosystems, highlighting the link between surface climate and subsurface biodiversity. DNA was extracted from granitic subsurface rocks recovered from two endmember sites along a north–south climate gradient in Chile’s Coastal Cordillera. Microbial communities inhabiting down to 55 m deep subsurface rocks were characterized using 16S rRNA amplicon and shotgun metagenomic sequencing. We identified an abundant and potentially active subsurface community below both climates dominated by heterotrophic bacteria, including *Pseudarthrobacter*, *Janthinobacterium*, and *Pseudomonas.* However, rare taxa affiliated with common chemolithoautrophs, e.g. *Thiobacillus*, *Sulfuriferula*, and *Sulfuricurvum*, were only observed in the arid subsurface, indicating increased oligotrophic conditions and reliance on inorganic electron donors in the deeper subsurface of the desert. Functional analysis revealed sulphur, hydrogen, and carbon monoxide as potential inorganic electron donors. These findings expand the current understanding of microbial life in the subsurface of granite rocks showing the influence of surface climate on nutrient conditions in the deeper subsurface, providing new insights into the extent and functional capacity of terrestrial subsurface habitats and their role in global biogeochemical processes.

## Introduction

Microorganisms inhabiting Earth’s subsurface within the pore space of sedimentary and magmatic rocks constitute a significant proportion of the planet’s biomass [1–4]. Within this realm known as the deep biosphere, microbial life adapts to various challenges, including anoxic, oligotrophic, and xeric conditions [5, 6], often exhibiting slow metabolic rates or dormant states to persist in these extreme habitats [7]. Despite being limited in nutrients and energy sources, these specialized microbial communities play a crucial role as biogeochemical agents in the subsurface, driving element cycling and potentially contributing to rock weathering [8, 9]. Given the expanse of deep biosphere ecosystems, this hidden microbial subsurface activity could significantly impact life-sustaining processes within the critical zone, the part of Earth’s crust where essential processes between lithosphere, hydrosphere, atmosphere, and biosphere lay the foundation for life on our planet [10].

Deep biosphere systems have been explored in both marine [11, 12] and terrestrial settings [13, 14], each exhibiting distinct characteristics. The terrestrial deep biosphere is part of the continental crust and comprises a greater variety of geological settings offering a more diverse array of habitats and conditions for microbial life than the marine deep biosphere [15]. Furthermore, fluid flow and habitat connectivity differ significantly between marine and terrestrial environments. In marine settings, chemical exchange is enhanced through molecular diffusion and the advection of fluids through subseafloor sediments and basaltic basement [16]. In contrast, fluid flow in terrestrial environments is primarily controlled by the transport of fluids along pore spaces and fractures originating from the surface or by the presence of oligotrophic deep aquifers [17–19]. Thus, when these environments extend deeper, they become increasingly isolated from surface-derived water, nutrients, and energy sources [6, 8, 18, 20]. Granitic rocks of the terrestrial deep biosphere typically crystallize at temperatures between 600 and 900°C and thus contain neither life nor any initial organics. Therefore, microorganisms have to colonize these subsurface environments through geological produced fractures that represent a connection between surface biomass and the igneous deep biosphere. Life within these rocks relies on the input of photosynthetically derived organic matter from the surface via fluid transport along fractures or the presence of chemolithoautotrophic primary producers [21], which can utilize gases such as H_2_ or CO_2_ and mineral components (e.g. S or Fe) for their energy and carbon supply [6, 22]. These chemolithoautotrophs have been shown to contribute directly to weathering processes in subsurface granitic rocks through the oxidation of mineral-derived iron [23, 24]. However, more common than direct redox reactions are indirect weathering processes of heterotrophs, including the production of acids [25] or siderophores [26, 27], which further accelerate chemical dissolution of the rock [28].

Although the scientific community’s interest in these challenging-to-access environments is growing, characterizing deep biosphere ecosystems, especially those underneath terrestrial environments, is extremely difficult [15, 29]. For more accessible sampling and contamination tracking, most studies on the terrestrial deep biosphere focus on the molecular analysis of recovered groundwaters [18, 30, 31], with only a few studies examining actual rock material [13, 32, 33]. Consequently, knowledge about desertic subsurface environments, where groundwater is non-existent or occurs deep below the surface, is still missing, and it is unknown whether microorganisms can persist in deep biosphere rocks below arid landscapes without regular water and nutrient supply from surface environments.

This study analysed microbial DNA extracted from granitic rock at depths of 1.5–55 m, recovered from drilling campaigns at two endmember sites along a north–south transect in Chile’s Coastal Cordillera, representing arid and humid surface conditions. By comparing 16S rRNA amplicons and shotgun metagenomes of the two ecosystems, we aimed to gain insight into whether microbial communities in these igneous subsurface environments are influenced by climatic conditions on the surface. We hypothesize that the limited infiltration of surface fluids in arid landscapes leads to stronger taxonomic and functional signals of chemolithoautotrophic metabolism compared to the humid subsurface. To further investigate the significance of those ecosystems for Earth system dynamics, we also screened the metagenomes for microbial weathering processes, including genes related to acidification, chelation, and biofilm formation, to assess whether these subsurface communities could contribute to deep weathering. Integrating all these data provided novel insights into microbial communities that inhabit subsurface granite rocks underneath a desert ecosystem. Comparing this community to its humid equivalent on a taxonomic and functional level not only allows us to directly characterize specific traits of subsurface ecosystems but also to anticipate their potential role within the critical zone.

## Material and methods

### Study sites and lithology

**Figure 1:**
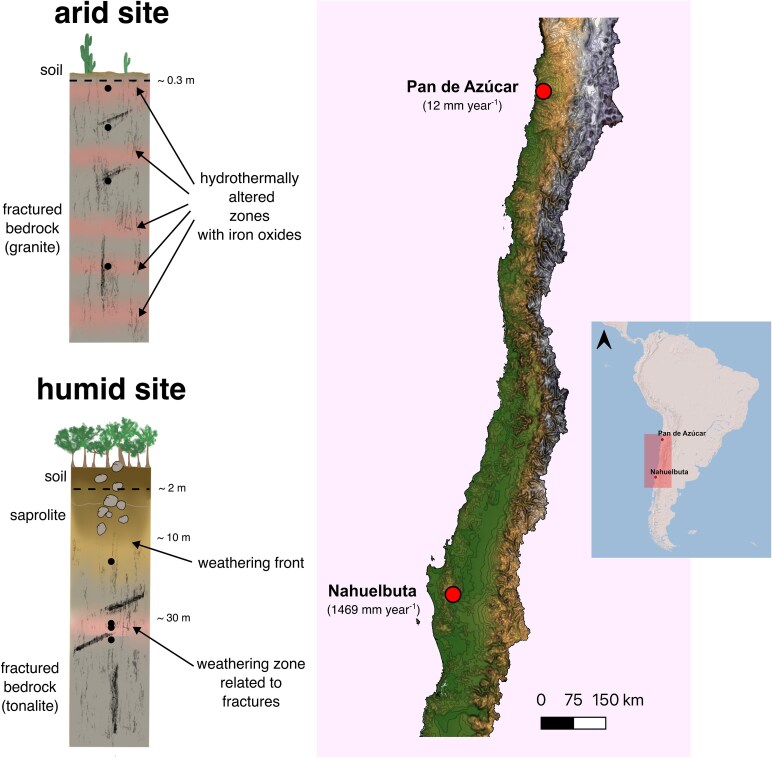
Drilling locations and schematic overviews of the rock cores show the main characteristics of the depth profiles, with dots marking sampling points, mean annual precipitation given in brackets, and exact sampling depths listed in Table 1.

Humid and arid subsurface samples were recovered during November 2019 and February 2020 in the framework of the Deep EarthShape project (DFG SPP 1803). This research project investigated deep weathering processes by drilling cores across a climate gradient reaching the unweathered bedrock. The goal was to explore the lowest extent of the critical zone and better understand interactions between the lithosphere and all atmospheric, hydrospheric, and biospheric processes. The project included four drilling sites within a north–south transect along the Chilean Coastal Cordillera. The transect spanned from arid conditions in the north to humid conditions in the south, maintaining a consistent granitic bedrock lithology. Further site characteristics were reported elsewhere [34–36]. In this study, we employed molecular biological methods on selected samples of the two end members of the transect, i.e. with the Pan de Azúcar site representing arid conditions and the Nahuelbuta site representing humid conditions (Fig. 1 and Supplementary Fig. S1). At the arid site in Pan de Azúcar, the lithology is granitic representing a highly fractured bedrock that shows signs of hydrothermal alterations in the form of secondary minerals such as iron oxides, calcite, and clays [37]. The humid site in Nahuelbuta has a slightly different lithologic composition being classified as a tonalite [35]. Iron oxides on the fracture surfaces of this tonalite can be attributed to chemical weathering, which is especially expressed in a weathering zone at 30 m depth. Rock core samples taken from these sites were typically 10–30 cm long and included a fracture surface with iron oxides.

**Table 1:** Overview of the analysed samples indicating their depth, location, and climate; for further information on the locations, see Fig. 1.

**Climate and mean annual precipitation**	**Sample ID**	**Depth**	**Site**	**Borehole coordinates and elevation**
**Arid** 12 mm yr^−1^	AZ 3	1.58–1.66 m	Pan de Azúcar	−26.302717, −70.457350, 732 m
	AZ 11	11.80–11.90 m		
	AZ 24	25.80–25.96 m		
	AZ 46	54.70–54.80 m		
**Humid** 1469 mm yr^−1^	NA 29	19.17–19.25 m	Nahuelbuta	−37.79381, −72.95043; 1114 m
	NA 39.0	30.30–30.48 m		
	NA 39.18	30.48–30.78 m		
	NA 41	33.66–33.72 m		

### Drill core sampling

The drilling was performed according to previously described methods [38]. Detailed description of the drilling procedure can be found in the Supplementary Material (S1.1). To minimize contamination, the outer core segments (2–3 cm) were removed using an aseptically cleaned mechanical rock trimmer. The inner portion was used for molecular analysis and stored at −20°C. Subsequently, samples were ground to a grain size of <2 mm using a flame-sterilized disk mill.

### Drilling contamination assessment

Concentrations of the contamination tracer (see Supplementary Material S1.1) were quantified for drilling fluid samples dripping down from wireline coring equipment while retrieving those core sections used for this study. Particle concentrations were quantified for subsamples of the cores using the same material used for DNA extractions. Subsequently, particle concentrations were converted to the volume of drill fluid infiltration per mass of material. Previously described methods were used for sample preparations, particle quantification, and calculation of contaminations [38].

Additionally, DNA was extracted from samples of the water tank and the liner fluid used during drilling. For these controls, liner fluid from each core run and daily water samples from the tank utilized for liner fluid preparation were collected in 50 ml Falcon tubes. After allowing particles to settle, the fluid was filtered onto a 0.22 μm Sterivex filter which was then stored at −20°C. DNA was extracted directly from the filter using a method previously applied on the same kind of filter during intracellular DNA extraction [39]. Filters were mixed with cetyltrimethylammoniumbromid (CTAB) buffer, phenol-chloroform-isoamyl alcohol (25:24:1), and 10% Sodium dodecyl sulfate (SDS) [40] in a 2 ml screw cap tube together with zirconia beads (0.1 and 1 mm size). After bead beating (FastPrep-24™, 6 m s^−1^, 45 s) and centrifugation (10 min, 16 000 *g*, 4°C), the aqueous phase was purified with chloroform-isoamyl alcohol (24:1) and guanidine HCl buffer [39]. DNA was then recovered by adding the mixture to silica spin columns and subsequent centrifugation (5000 *g* for 1 min). The column was washed twice by adding a washing buffer [39, 41] and subsequent centrifugation (5000 *g*, 1 min). DNA was eluted with polymerase chain reaction (PCR)-grade water and stored at −20°C. For library preparation and 16S rRNA sequencing, drill control (liner fluid) and water tank controls were treated similarly to samples while sequenced in separate libraries. PCR cycles were increased to 35 cycles to ensure sufficient DNA recovery.

### Geochemical analysis

Water-leachable ions, pH, and electrical conductivity were measured from powdered rock leachates (1:5 w/v rock to Milli-Q water; Supplementary Material S1.2). Ion chromatography was used to quantify major cations and anions, while pH and electric conductivity (EC) were measured with a pH metre and a conductivity probe from the same leachates.

### Enzymatic analysis

Microbial activity was assessed via a fluorescein diacetate (FDA) hydrolysis assay [42] (Supplementary Material S1.3). Rock powder was incubated with the FDA solution, and fluorescein release was quantified spectrophotometrically at 490 nm.

### 16S rRNA gene analysis

#### DNA extraction

DNA was extracted under sterile conditions in a clean lab using a modified protocol based on the phenol-chloroform method [40]. Approximately 1 g of ground rock was added to bead tubes with zirconia beads and a sodium phosphate-ethylenediaminetetraacetic acid (EDTA) buffer (300 mM of Na_2_HPO_4_, NaH_2_PO_4,_ and EDTA; pH 8) [39], followed by 10% SDS. Samples were subjected to freeze–thaw cycles (−80/900°C), bead beating (6 m s^−1^, 45 s), and enzymatic lysis with Proteinase K (5 μl of 0.5 mg ml^−1^). Lysates were mixed with 600 μl phenol-chloroform-isoamyl alcohol (25:24:1), centrifuged (16 000 *g*, 10 min, 4°C), and purified with 600 μl of chloroform-isoamyl alcohol (24:1) and guanidine HCl buffer [39]. DNA was bound to silica columns, washed, and eluted in PCR-grade water. For each sample, nine replicates were extracted, of which three replicates were pooled during the silica binding step of the extraction for better DNA recovery. Negative extraction controls were treated identically.

#### Quantitative PCR

Quantitative polymerase chain reaction (qPCR) was performed using a CFX Connect Real-Time PCR detection system (Bio-Rad) with KAPPA HiFi SYBR Mix 1x (Qiagen) and universal prokaryotic primers 341F (5′-CCTACGGGAGGCAGCAG-3′) and 534R (5′-ATTACCGCGGCTGCTGG-3′) [43]. The cycling parameters were used as described before [39], and samples were analysed by running three technical replicates. A 16S rRNA gene fragment of *Bacillus subtilis* (2.5 × 10^8^ gene copies) was used to generate a standard curve via serial dilution (10^1^–10^7^ gene copies) and to calculate the efficiency (>90% to <110%). All samples with a cq value below the one of the no-template control (cq = 31) were excluded from the analysis.

#### Amplicon sequencing

The 16S rRNA V4 region was targeted with primers F′515 (5′-GTGYCAGCMGCCGCGGTAA-3′) [44]; and R′806 (5′-GGACTACNVGGGTWTCTAAT-3′) [45]. PCR with barcoded primers was done using the following programme: 5 min denaturation step at 95°C followed by 31 cycles of 30 s at 95°C, 30 s annealing at 56°C, and 1 min extension at 72°C. Illumina paired-end sequencing was on an Illumina HiSeq machine with MiSeq V3 chemistry (2 × 300 bp paired-end reads). The sequencing library was demultiplexed using cutadapt v3.5 [46] using the following parameters: -e 0.2 -q 15,15 -m 150 --discard-untrimmed. Amplicon sequence variants (ASVs) were generated from trimmed reads with the DADA2 package v1.20 [47] with R v4.1 using the pooled approach with the following parameters: truncLen = c(240,200), maxN = 0, rm.phix = TRUE, minLen = 200. Taxonomic assignment was done using DADA2 and the SILVA database v138 [48]. Lab contaminants were identified based on their presence in extraction and PCR controls and removed together with non-target ASVs (chloroplasts, mitochondria, and singletons). For diversity analysis, samples were rarefied to 1299 reads using the vegan package [49] in R. Class- and ASV-level visualizations were based on relative abundances from non-rarefied data. Network analysis was performed on a normalized, abundance-filtered ASV table (≥0.1% mean abundance), using Spearman correlations (cut-offs: ρ ≥ 0.6, *P* ≤ .1), and visualized with igraph [50]. All plots were generated using phyloseq, vegan, and ggplot2 [49, 51, 52].

### Shotgun metagenomics

DNA extraction for metagenomic sequencing was identical to the extraction protocol used for 16S rRNA gene analysis. Each sample was extracted using 18 replicates plus three negative controls containing no sample material for shotgun metagenomic sequencing. The pooled negative control yielded no measurable or amplifiable DNA (for PCR specifications, see the section “16S rRNA - amplicon sequencing” above). Shotgun metagenomics sequencing was performed by CeGaT Germany (Tübingen, Germany) using an Illumina NovaSeq and 2 × 150 bp paired-end reads. Data processing was done using the ATLAS pipeline v2.12.0 [53], including *metaSPAdes* v3.15.3 [54] for read assembly into contig, *eggNOG mapper* v2.1, and *eggNOG database* v5.0 for functional gene annotation [55]. Default parameters were used. For functional analysis, the ATLAS output was screened for key genes involved in different pathways, weathering mechanisms [56] and adaptation mechanisms based on the Kyoto Encyclopedia of Genes and Genomes (KEGG) Orthology (Supplementary Tables S1 and S2). Gene abundance was transformed into reads per million (rpm) and visualized using the *heatmap.2* function of the *gplots* [57] package in R. Iron-related genes in the metagenomic dataset were investigated using the *FeGenie* package [58].

## Results

### Drilling contamination

Concentrations of the tracer ranged from 8.3 × 10^10^ to 1.2 × 10^12^ particles l^−1^ in the drilling fluids of Pan de Azúcar and from 7.0 × 10^10^ to 2.8 × 10^11^ particles l^−1^ in the drilling fluids of Nahuelbuta. Tracer concentrations in the rock material ranged from 8.5 × 10^4^ to 3.6 × 10^5^ g^−1^ rock in Pan de Azúcar and from 1.0 × 10^5^ to 8.9 × 10^5^ g^−1^ rock in Nahuelbuta. Based on these values, we can show that minor amounts of up to 4.3 μl g^−1^ of drilling fluid entered the inner part of the rock core (Supplementary Table S3). Additionally, comparison of the 16S rRNA data recovered from the water tank and the liner fluids with the actual rock core samples showed that drill controls and water tank control did not cluster together with the analysed samples, inferring that the injection of liner fluid had not disturbed the *in situ* communities (Supplementary Fig. S2A and B).

### Geochemistry

Soluble ion concentrations were generally higher in the arid than the humid subsurface, with sodium, chloride, and sulphate ions dominating and showing a decline with depth in the arid profile. In contrast, concentrations in the humid profile were consistently low (Supplementary Material S2.1, Supplementary Fig. S3, Supplementary Table S4). pH values were generally high, ranging from 7.6 in the shallow arid horizon to 9.4 in deeper arid samples, while humid horizons ranged from 7.9 to 8.6. Electrical conductivity was an order of magnitude higher in arid samples (93–130 μS cm^−1^) compared to humid ones (8–14 μS cm^−1^; Supplementary Fig. S4, Supplementary Table S5).

### Microbial activity

FDA hydrolysis assays revealed measurable microbial activity in most samples, with higher activities at depth in the humid subsurface and detectable, though lower, activity in the deeper arid subsurface samples (Supplementary Material S2.2, Supplementary Fig. S5, Supplementary Table S6). For the two shallow arid subsurface samples at 1.5 and 12 m depth, activity was below the detection limit.

### 16S rRNA abundance and sequencing results

Overall, 16S rRNA gene sequencing of the eight core samples generated 399 009 reads, identifying 1742 unique ASVs. The removal of low-quality sequences, mitochondria, chloroplasts, and potential lab contaminants reduced sequencing depth and ASV number to 248 693 remaining reads distributed across 1156 different ASVs. Ninety-eight percent of the ASVs were assigned to bacterial taxa, and the remaining 2% were assigned to archaeal taxa.

**Figure 2:**
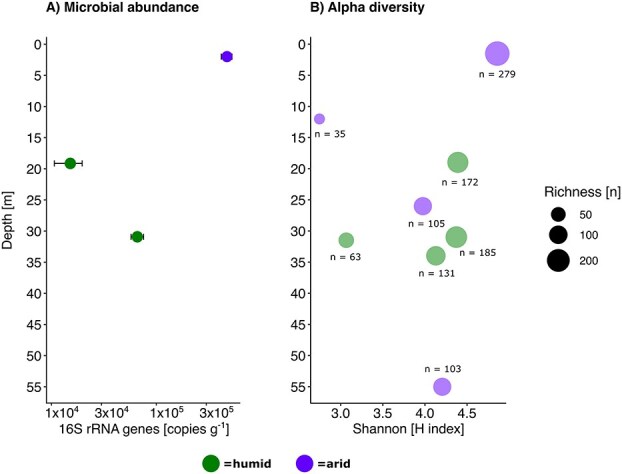
Microbial abundance (16S rRNA gene copy numbers from qPCR; x-axis on a logarithmic scale) and alpha diversity (subsampled ASV richness [n] as bubble size and Shannon index [H] on the x-axis) across different depths in the arid and humid subsurface environments.

Due to the low biomass, microbial abundance using qPCR could only be assessed in three samples across both cores (Fig. 2A). In the arid subsurface, the 1.5 m deep sample yielded 4.7 × 10^5^ gene copies g^−1^ rock (Supplementary Table S7). All other arid samples were not quantifiable. In the humid subsurface, two samples were quantifiable, i.e. from 19 to 31 m depth with gene copy numbers g^−1^ rock of 1.5 × 10^4^ and 6.6 × 10^4^, respectively.

Diversity was generally low, with Shannon indices following richness patterns (Fig. 2B). The highest diversity was observed for the shallow arid sample at 1.5 m depth with a Shannon index of 4.9 and 279 observed ASVs (Supplementary Table S7). At 12 m depth, diversity was even lower, with Shannon indices of 2.7 and only 35 observed ASVs. Diversity was higher for the two deeper samples at 26 and 55 m, as Shannon indices of 4.0 and 4.2 and richness values of 105 and 103 were determined. In the humid subsurface, the highest diversity was observed for the two samples at 19 and 31 m depth, with Shannon values of 4.4 and richness values of around 180 observed ASVs. At 31.5 m depth, the sample below exhibited the lowest diversity in the humid subsurface with a Shannon value of 3.1 and 63 observed ASVs. At 34 m depth, diversity was again slightly higher, showing a Shannon value of 4.1 and 131 observed ASVs.

### Microbial community composition

Almost 90% of the overall microbial abundance could be attributed to four phyla, i.e. *Proteobacteria* (50.4%), *Actinobacteriota* (25.6%), *Firmicutes* (8.1%), and *Bacteroidota* (4.9%) (Supplementary Table S8). Most arid and humid samples were dominated by the *Proteobacteria* classes *Gamma*- and *Alphaproteobacteria* (Supplementary Fig. S6). Generally, the phylum *Actinobacteriota* was more abundant in the humid samples. *Actinobacteriota* communities at both sites were dominated by *Actinobacteria*, with relative abundances of 3.2%–16.5% in the arid subsurface and 19.2%–55.0% in the humid subsurface. The two shallow arid samples (1.5 and 12 m) were also characterized by *Thermoleophilia* and the *Actinobacteriota* class MB-A2-108, with relative abundances of 16.2% and 4.9% at 1.5 m and 4.5% and 3.3% at 12 m depth, respectively. *Firmicutes* were frequently detected in the arid samples, most of which were assigned to the class *Bacilli*, ranging in relative abundance between 6.2% and 16.9% (Supplementary Fig. S6). The relative abundance of *Bacilli* decreased in humid samples from 7% at 19 m depth to 1.2% and 2.6% at 31 and 31.5 m depth. At a depth of 34 m, no *Bacilli* signatures could be observed.

**Figure 3:**
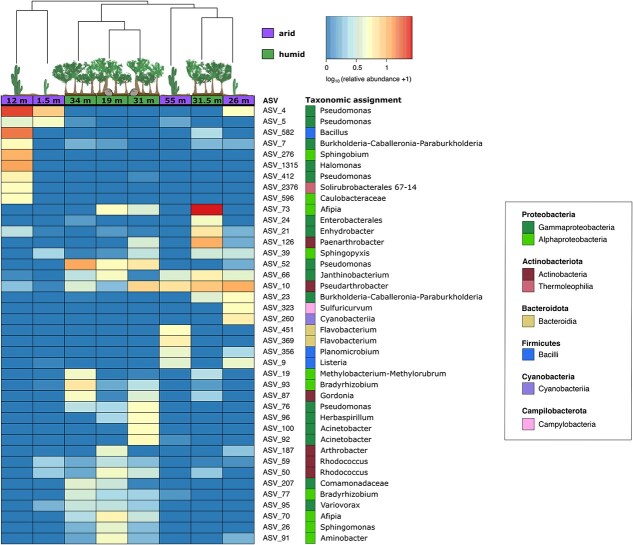
Heatmap showing the most abundant ASVs (mean relative abundance >0.5%) with log10-transformed relative abundance values, taxonomic assignments, and samples ordered by average-linkage hierarchical clustering of Bray–Curtis dissimilarity.

The evaluation of the most abundant ASVs revealed two main clusters of samples (Fig. 3). One cluster includes the two shallow arid samples from 1.5 to 12 m depth, and the other all other deeper samples from the arid and humid subsurface. The specific arid cluster is characterized by two abundant *Pseudomonas* ASVs, i.e. ASV_4 (3.8% and 7.7% relative abundance) and ASV_5 (2.9% and 19.8% relative abundance; Fig. 3, Supplementary Fig. S9 & S10). The other ASVs of the arid cluster were dominant in only one of the two samples. The 1.5 m deep sample included ASV assigned to *Sphingopyxis* (ASV_39; 1.2%), *Rhodococcus* (ASV_50; 1.1%, and ASV_59; 1.1%), and *Variovorax* (ASV_95; 0.7%). The 12 m sample included ASVs of *Bacillus* (ASV_582; 14.7%), *Halomonas* (ASV_1315; 11.3%), *Burkholderia–Caballeronia–Paraburkholderia* (ASV_7; 4.4%), and *Solirubrobacterales* 67-14 (ASV_2376; 4.3%). The cluster comprising the deeper samples from the arid subsurface (26 and 55 m) and all humid samples was defined by the occurrence of ASV_10 *Pseudarthrobacter* (0.8–10.6%) and ASV_66 *Janthinobacterium* (0.1%–5%). Within the cluster, we also observed several subclusters. One subcluster included the 19 , 31 , and 34 m deep samples, which included ASV_52 and ASV_76 assigned to *Pseudomonas* (1.3%–10.9%) and an *Afipia* ASV_73 (1.5%–4.7%). The other subcluster included the 26 and 55 m deep arid samples and the 31.5 m deep humid sample. It was primarily characterized by ASV_10 *Pseudarthrobacter* (5.9%–10.8%) and ASV_66 *Janthinobacterium* (2.6%–5.0%). These two ASVs were the only ones shared by all three samples of the latter subcluster. The ASV_73 Afipia and ASV_126 *Paenarthrobacter* were predominant (25.8% and 10.4%) in the 31 m deep, humid sample; the ASV_323 *Sulfuricurvum* and ASV_260 *Cyanobacteria* were found exclusively in the 26 m deep arid sample and with comparatively high abundances (4.3% and 5.1%). Similarly, the two *Flavobacterium* ASVs, ASV_451 (4.2%) and ASV_369 (5%), were retrieved exclusively from the 55 m deep arid sample.

**Figure 4:**
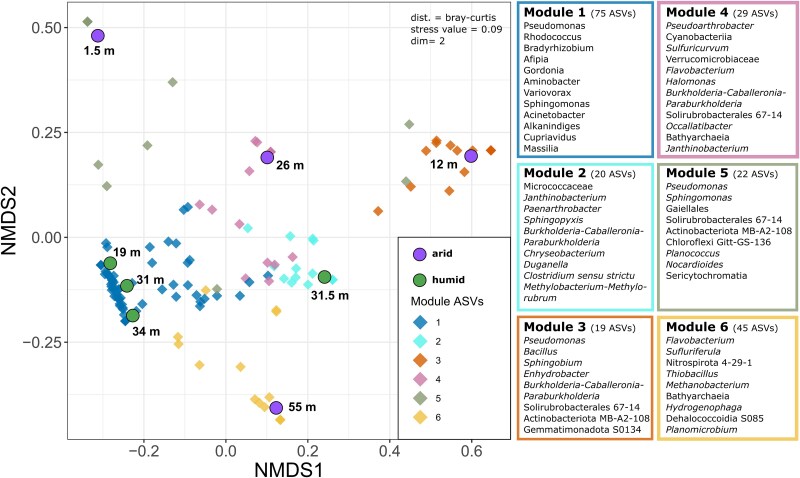
Non-metric multidimensional scaling (NMDS) plot showing sample dissimilarities, with ASVs colored by their module assignment in a co-occurrence network (ASVs with >0.1% mean relative abundance, correlation cut-off 0.6, and P-value cut-off 0.1), and representative ASVs from each module listed on the right (complete list of ASVs and their relative abundances in Supplementary Table S15).

Co-occurrence network analysis of the ASVs (>0.1 mean relative abundance across all samples; 210 ASVs overall) revealed six different modules of ASVs that co-occur (Supplementary Fig. S7). These ASVs are indicated in an Non-metric multidimensional scaling (NMDS) plot representing sample dissimilarities, thus showing the sample association of each module (Fig. 4). Modules 1 and 2 were mainly characterized by ASVs which co-occur in the humid subsurface with module 1 representing taxa associated with the 19 , 31 and 34 deep samples and module 2 taxa being associated with the 31.5 m deep sample. ASVs of this module were mainly present in the most abundant ASVs displayed in Fig. 3, including ASVs affiliated to *Bradyrhizobium*, *Burkholderia-Caballeronia-Paraburkholderia*, *Janthinobacterium*, and *Pseudomonas.*

The modules indicative of arid samples were closely associated with single samples and contained more specific ASVs, which did not occur in the most abundant ASVs. Module 3 ASVs are mainly associated with the 12 m deep arid sample and encompassed ASVs such as *Actinobacteriota* MB-A2-108 and *Chthoniobacter*. Module 4, representing the 26 m deep arid sample, also contained *Actinobacteriota* MB-A2-108 and ASVs of *Bathyarchaeia* and *Cyanobacteria*. The shallow arid sample was represented by Module 5, including *Actinobacteriota* taxa assigned to *Actinobacteriota* MB-A2-108, *Chloroflexi* Gitt-GS-136, *Sericytochromatia* (*Cyanobacteria*), *Gaiellales*, and *Nocardioides*. Module 6 indicated the composition of the deepest sample in the arid subsurface recovered from 55 m depth. This module was defined by a variety of specific ASVs, including *Actinobacteriota* MB-A2-108, *Bathyarchaeia*, *Dehalococcoidia* S085, *Hydrogenophaga*, *Methanobacterium*, *Nitrospirota* 4-29-1, *Paenisporosarcina*, *Sulfuriferula*, and *Thiobacillus*.

### Metabolic potential of subsurface communities

To assess the genetic potential of the subsurface communities, shotgun metagenomic data of samples from the humid subsurface (31 m depth) and arid subsurface (1.5 and 26 m depth) were analysed. After dereplication and quality control, 20, 35, and 13 mio reads, respectively, remained (for details see Supplementary Table S14). After assembly, the total combined length of all contigs from all three samples was 216 mio bp and 229 247 different genes were detected, which were annotated and screened for key genes related to redox reactions, carbon, and nitrogen fixation.

**Figure 5:**
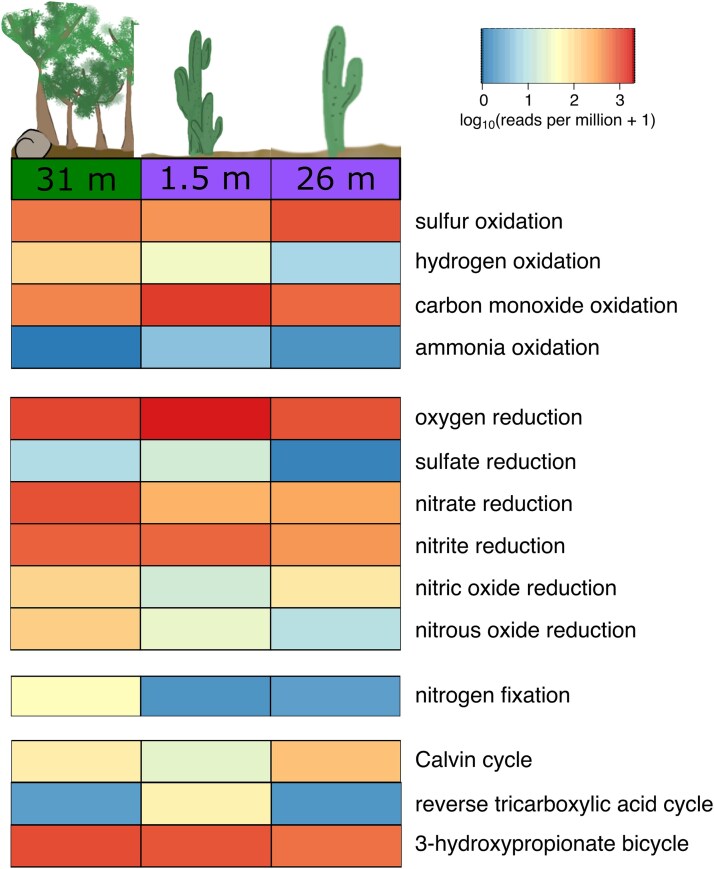
Heatmap showing the relative abundance (log10-transformed rpm) of selected functions (Supplementary Table S1) across the three metagenomic datasets (for untransformed rpm values, see Supplementary Table S11).

In terms of potential electron acceptors, genes related to the reduction of oxygen (*coxAC*), nitrate (*narBGHYZ*, *napAB*), and nitrite (*nrfAD*, *nirBDK*) occurred abundantly throughout all metagenomics samples (Fig. 5). Oxygen reduction genes were most abundant in the shallow arid sample, showing 1344 rpm, while they were half as abundant in the two deeper samples from the arid and humid subsurface (Fig. 5; Supplementary Table S11). Nitrate reduction genes appeared to be most abundant in the humid subsurface, showing 680 rpm compared to around 200 rpm in both arid samples. Genes related to nitrite reduction are also most abundant in the humid sample with 560 rpm, closely followed by the shallow arid sample with 527 rpm and 290 rpm for the deep arid sample. Less abundant were genes associated with the reduction of nitric oxide (*norBC*), nitrous oxide (*nosZ*), and sulphate (*dsrABC*, *aprAB*). While nitric and nitrous oxide reduction were most abundant in the humid sample (93 and 106 rpm), sulphate reduction genes were highest in the 1.5 m deep arid sample with 13 rpm. No genes related to the reduction of iron were observed using the FeGenie pipeline (Supplementary Table S12).

The ability to oxidize inorganic compounds can be an important trait for subsurface organisms to survive without photosynthetically produced organic matter. Analysis of the genetic potential revealed genes involved in the oxidation of sulphur (*fccAB*, *sqr*, *soxABXY*, *soeABC*, *tcdH*, and *scnABC*) and carbon monoxide (*coxG*, *coxM*, and *coxS*) to be abundant across all samples. The values for sulphur oxidation ranged from 296 to 654 rpm, while carbon monoxide oxidation ranged from 359 to 867 rpm. Genetic potential for hydrogen oxidation (*hydABC*, *hyaBCD*, *hybCD*, ferredoxin hydrogenase small subunit) was highest in the humid subsurface with 89 rpm, whereas hydrogenase abundance decreased with depth from 27 to 5 rpm in the arid subsurface.

Few genes for ammonia oxidation (*amoA*) were detected, with only 3 rpm observed in the 1.5 m deep arid sample and 1 rpm in the 26 m deep arid sample. In the humid subsurface, ammonia oxidation genes were not detectable. Minimal evidence was also found for the oxidation of iron, as only 1 count per million (cpm) was detected in the 31 m deep humid and 1.5 m arid samples (Supplementary Table S12). In the 26 m deep arid sample, no genetic potential for iron oxidation was found.

The potential of chemolithoautotrophic growth was assessed by screening key genes for different carbon fixation pathways. These pathways showed a mostly even distribution across all three metagenomes. Calvin cycle (*PRK*, *rbcLS*) and 3-hydroxypropionate bicycle (*accABCD*)-associated genes were identified at an abundance of 20–140 rpm and 453–707 rpm. Genes associated with the reverse tricarboxylic acid cycle (*ccsA*) did not appear abundantly, exhibiting the highest value of 46 rpm in the 1.5 m deep arid sample and significantly less in the other two samples. Genetic markers for the Wood–Ljungdahl pathway (*cdhD*) were not detected across all samples. The ability of nitrogen fixation was investigated by screening for the *nifH* gene. This gene showed the highest abundance of 38 rpm in the humid subsurface while being very low in the two arid samples, showing 0.7 and 1.1 rpm at 1.5 and 26 m depth.

### Microbial weathering potential and survival strategies

**Figure 6:**
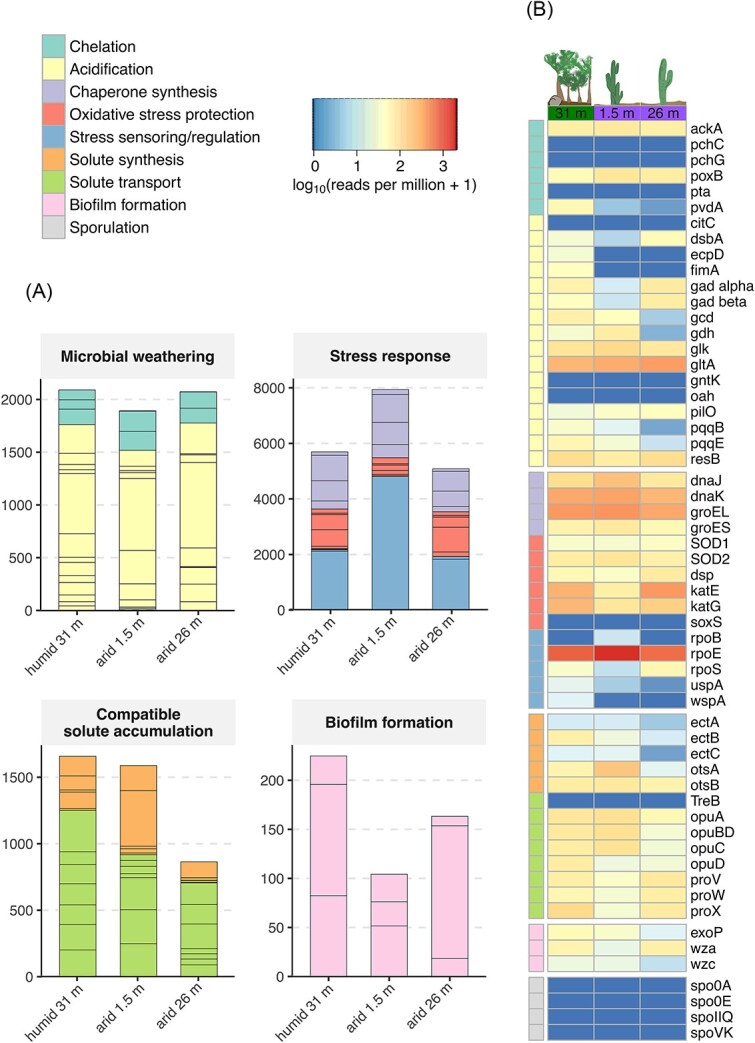
Distribution of genes related to microbial weathering, adaptation to xeric stress, biofilm formation, and sporulation in the metagenomic datasets, with summed rpm values for functional categories shown in barplots (A) and corresponding individual genes presented as log10-transformed rpm in a heatmap (B).

To assess the weathering potential and adaptations to nutrient-limited and dry conditions, the metagenomes were tested for key genes involved in microbial weathering, stress response, compatible solute accumulation, biofilm formation, and sporulation (Fig. 6).

Acidification and chelation genes, previously reported to contribute to weathering processes [56], were found to be equally abundant in all three metagenomes (Fig. 6A). Genes associated with acidification accounted for nearly 1800 rpm in the deep humid and arid subsurface, while the shallow arid sample showed a slightly lower abundance of around 1500 rpm. Among these, gltA (citrate synthase) and glk (glucokinase) were consistently abundant across all samples (Fig. 6B). In contrast, genes such as *gcd* (quinoprotein glucose dehydrogenase), *ghd* (glucose dehydrogenase), *gad* (gluconate 2-dehydrogenase), as well as the pyrroloquinoline quinone biosynthesis genes (*pqqB*, *pqqE*), displayed variable abundances among the three subsurface environments. The three chelation genes detected in our dataset were *ackA* (acetate kinase), *poxB* (pyruvate dehydrogenase (quinone)) and *pvdA* (L-ornithine N5-monooxygenase), together showing 374 rpm at 1.5 m depth of the arid subsurface, 296 rpm at 26 m depth of the arid subsurface, and 327 rpm at 31 m depth of the humid subsurface (Supplementary Table S13). Gene counts for siderophore-related processes were also identified in all samples using the FeGenie pipeline, with siderophore synthesis genes showing 9–22 cpm, siderophore transport genes showing 47–189 cpm, and siderophore transport potential genes showing 22–69 cpm (Supplementary Table S12).

Genes associated with environmental stress were highest in the shallow arid sample at 1.5 m depth counting nearly 8000 rpm, compared to 5700 rpm in the 31 m deep humid and 5100 rpm in the deeper arid subsurface at 26 m depth. This was mainly facilitated by the higher abundance of the envelope stress regulator (*rpoE*), which, with 4800 reads, was more than twice as abundant in the 1.5 m arid subsurface than in the other two subsurface horizons. In contrast, genes that encode for oxidative stress responses, such as the reactive oxygen species (ROS) scavenger katE and katG, showed higher abundance in the deep subsurface 26 m below the arid site and 31 m beneath the humid site. Chaperone synthesis genes (*dnaJK*, *groEL*, *groES*) were also highest in the 1.5 m deep arid subsurface with an abundance of nearly 2500 rpm compared to 1500 rpm in the deeper 26 m arid subsurface and 2000 rpm in the humid subsurface at 31 m depth.

Genes involved in the accumulation of compatible solutes were more abundant in the 31 m humid and the 1.5 m arid subsurface (~1600 rpm) than in the deeper arid subsurface at 26 m (~900 rpm). The high abundance of these genes in the 31 m deep humid subsurface was mainly attributed to a strong presence of solute transport genes (*opuABCD*, *proVWX*), while in the 1.5 m arid subsurface, genes for trehalose synthesis (*otsA*, *otsB*) were specifically enriched relative to both deeper arid and humid subsurface samples. The deep arid subsurface at 26 m showed generally the lowest abundances for both solute synthesis (~160 rpm) and transport (~700 rpm).

Biofilm formation genes were highest in the 31 m deep humid subsurface (225 rpm), followed by the 26 m deep arid subsurface (163 rpm), and least abundant in the 1.5 m deep subsurface (104 rpm). Notably, the gene encoding the polysaccharide biosynthesis/export protein *wza* was particularly depleted in the shallow 1.5 m arid subsurface. Genes associated with sporulation (*spoOA*, *spolE*, *spollQ*, *spoVK*) are absent in all three metagenomes.

## Discussion

In this study, we characterized microbial community composition, potential energy sources, potential roles in weathering processes, and adaptation strategies within humid and arid granitic subsurface environments, down to a depth of 55 m. We employed a combination of 16S rRNA sequencing and shotgun metagenomics to reveal differences in the community composition and functional potential between the two deep subsurface environments, which may be influenced by the different climate conditions on the surface.

Analysis of microbial community composition indicated two main clusters, one representing the 1.5 and the 12 m deep arid subsurface, and the other all depths below 19 m from both arid and humid subsurface. Besides dominant *Proteobacteria* populations, including several *Pseudomonas* ASVs, the two shallow arid environments comprised several taxa, which are common to desert soils. This is particularly reflected in the presence of *Thermoleophilia* and the unclassified class MB-A2-108, both *Actinobacteriota* classes, which have been observed in desert environments, i.e. of arid and hyperarid soils of the Atacama Desert, before [59–61]. More specifically, a member of *Thermoleophilia* assigned to the unclassified family *Rubrobacterales* 67-14, which is characteristic of the shallow arid subsurface, has been identified as a specialist in communities within 2-m-deep soil profiles in the same arid region [62]. Our investigation also revealed members of other microbial phyla previously associated with desert environments. These included *Chloroflexi* unclassified class Gitt-GS-136 [63], observed in the 1.5 m deep arid sample, and *Gemmatimonadota* unclassified class S0134_terrestrial_group [64] in the 12 m deep arid sample. We conclude that the presence and number of highly adapted *Actinobacteriota* and other known desert and soil microorganisms in the shallow arid samples indicate the influence of desertic soil microbial communities. These findings suggest that the shallow arid subsurface (down to 12 m) is more connected to surface ecosystems being shaped by the dry surface conditions.

In contrast, desert soil-associated *Actinobacteriota* are less abundant in the deeper arid subsurface and absent in the humid subsurface, which together form the big cluster of all samples below 19 m depth. This big cluster was dominated by many aerobic bacteria, particularly assigned to *Proteobacteria*, reflecting previous findings in granitic deep biosphere environments [13, 14, 20, 65]. Two main ASVs belonging to the genera *Janthinobacterium* and *Pseudarthrobacter* were especially characteristic of these deeper microbial communities*. Janthinobacterium* is an aerobic and heterotrophic genus whose members can contribute to nitrogen cycling under oligotrophic conditions [66]. The genus has been detected in 430 m deep continental and marine subsurface samples [67, 68]. Furthermore, *Janthinobacterium* was found to be associated with rocks, having been isolated from granitic material of a glacier forefield [69] and basaltic material from the bedrock-soil interface [70]. The other genus, *Pseudarthrobacter*, is not considered a common deep biosphere inhabitant but has been observed in terrestrial subsurface sediments [71]. Similar to *Janthinobacterium*, *Pseudarthrobacter* has been found to inhabit granite-associated habitats [72]. *Pseudarthrobacter* is also widely distributed in terrestrial soils, ranging from Antarctic to desert environments, highlighting its high adaptation to environmental stressors such as oligotrophy, radiation, and different temperatures [73–75]. *Pseudomonas* ASVs were also detected in the deeper environments of this study. Those *Pseudomonas* ASVs differed from those found in the shallow arid subsurface. The same was observed for ASVs assigned to *Burkholderia-Caballeronia-Paraburkholderia*, a genus often found in soils [76], rhizospheres [77], and even polluted environments [78] and sediments [79]. Both *Pseudomonas* and members belonging to the order *Burkholderiales* have been commonly observed in terrestrial deep biosphere environments, showing versatile metabolic capabilities [31, 80–82]. Stress tolerance and adaptability are important prerequisites to inhabiting rock-associated and deep biosphere habitats [72, 80]. Furthermore, the flexibility allows them to inhabit a variety of environments. This wide distribution across different surface habitats is essential for colonizing the subsurface niches, as it increases the probability of being dispersed along fractures into deeper habitats. Thus, the genetic flexibility, together with the ubiquitous distribution of these taxa across different soils around the globe, may favour their strong presence in all the deeper (> 19 m deep) subsurface environments.

To assess the potential impact of surface climate on microbial subsurface communities beyond taxonomic shifts, the three metagenomes were analysed for genes involved in adaptation to xeric conditions, including universal stress responses, the accumulation of compatible solutes, and biofilm formation. Universal stress response genes suggest higher environmental stress in the 1.5 m deep arid subsurface than in the two deeper subsurface samples. This is mainly driven by the envelope stress regulator *rpoE*, which is known to be upregulated under xeric stress and in strains isolated from desert surface environments [83–85]. These findings indicate that the elevated stress at 1.5 m depth is likely linked to the dry conditions prevailing near the desert surface. This is further supported by genes associated with the synthesis of compatible solutes, particularly trehalose synthesis genes (*otsAB*), which are recognized indicators of desiccation stress in experiments and hyperarid environments [83, 86, 87], and were also most abundant in the 1.5 m arid subsurface. Although genes related to solute transport were not enriched in this horizon, it is known that the uptake of solutes is energetically more feasible than the synthesis [88]. We, therefore, argue that genes for the transport of solutes might be more widespread across microbial taxa and that the capacity to synthesize these osmoprotectants is a stronger signal of adaptation to xeric stress, consistent with the 1.5 m arid subsurface harbouring more desert taxa than the deeper arid subsurface at 26 m depth.

Other genetic adaptations are differently abundant across all three subsurface horizons, underlining that many of the same stress responses apply to different sources of environmental stress [85]. While genes for chaperone synthesis indicate no difference between the three metagenomes, oxidative stress response and biofilm formation genes appear to be more abundant in the deeper subsurface horizons of the humid and arid sites. Oxidative stress is known to occur during the formation of ROS in the absence of water [89]. However, subsurface processes such as radiolysis, radioactivity, mineral-water interactions could also produce ROS [6, 90]. All subsurface samples analysed in this study showed a strong presence of oxidation products on fracture surfaces that either originate from chemical weathering or hydrothermal alteration. Regardless of the process behind this, the presence of oxidation products in combination with a higher abundance of catalase genes (*katEG*) could infer that the rock-associated habitat in deeper subsurface environments applies more oxidative stress to the microbial community than the dry conditions at 1.5 m depth in the arid subsurface. Although biofilm formation can be an important process to compensate for xeric stress [85], it is also essential for subsurface communities to attach to rock and fracture surfaces and to retrieve nutrients from the mineralogy [91]. However, read sums for biofilm formation genes are relatively low, showing a maximum of 225 rpm in the 31 m deep humid subsurface. Therefore, biofilm formation like sporulation, where genes were entirely absent, might play a minor role due to energy limitations in these specific subsurface ecosystems.

It is important to note that the maximum depth analysed in this study (55 m for 16S rRNA sequencing and 31 m depth for shotgun metagenomics) is shallow compared to many other deep biosphere studies done on igneous bedrock. However, on the taxonomic and functional level, the two shallower arid horizons (1.5 and 12 m) still reflect taxonomic and functional traits of desert surface communities, while deeper humid and arid subsurface horizons (>19 m depth) align more closely with typical deep biosphere communities. Although these communities may not be directly comparable to those described from hundreds to thousands of metres depth, they already reveal similar ecological dynamics, potentially reflecting a shift from surface to deeper subsurface conditions. Thus, comparing these arid and humid “shallow deep biosphere” ecosystems provides valuable insight on how microbial communities and nutrient gradients are influenced by different climates on the surface.

The presence of primarily aerobic taxa and abundant genes related to oxygen reduction indicates that aerobic conditions prevail within the depth covered in our study (up to 55 m depth). Deep biosphere environments often become more anoxic with depth. However, the availability of oxygen mostly relies on respiration rates and the availability of reduced material, primarily in the form of organic matter [92]. Since igneous rocks lack inherent organic material and the subsurface habitat exhibits low biomass, atmospheric oxygen may diffuse down to the study’s maximum depth of 55 m. The presence of oxygen for most of the subsurface environments in this study is also reflected in the absence of many typical anaerobic deep biosphere organisms, such as dissimilatory sulphate reducers, including *Desulfotomaculum*, *Desulforudis*, and Candidatus *Desulforudis audaxviator* [13, 22, 93]. Only a few taxa encountered in this study are known as anaerobes. They include *Bathyarchaeia*, identified at 26 and 55 m depth underneath the arid site, and *Thermoanaerobacter* detected at 34 m depth underneath the humid site. Both taxa are known from deep biosphere studies. *Bathyarchaeia* found in deep groundwaters and subsurface sediments are known to yield the genetic potential for methanogenesis under anaerobic conditions [94, 95]. *Thermoanaerobacter* was also found in deep fracture fluids [96], and species of this genus have been proven to reduce a variety of different metals for anaerobic respiration [97]. The presence of these few anaerobic genera could indicate anoxic micro-niches in the pore space of the deeper subsurface. Key genes for the utilization of alternative electron acceptors to oxygen, especially nitrate and nitrite, were found in all three available metagenomes. This further underlines the metabolic flexibility of these subsurface microbial communities.

Microbial colonization of granitic subsurface rocks is controlled by the transport of cells from the surface. Despite the distinct rock-weathering-related deep biosphere composition which is shared between the arid and the humid subsurface, members of the class *Thermoleophilia* still occur in low abundances in the 26 m deep arid subsurface before they disappear in the 55 m deep arid subsurface. This gradual decrease of these desert soil taxa which are abundant in the 1.5 and 12 m deep subsurface could indicate the downward transport of microbial biomass into the subsurface. Due to the low porosity of the granitic rock material, it is expected that cell transport would occur through fluid flow along tectonic fractures during rare rain events in the Atacama. As the humid subsurface environments analysed in this study are starting from a depth of 19 m, it is difficult to conclude on the connectivity of these habitats to surface ecosystems. However, the humid subsurface environments show a high overlap of taxa, of which some are known to be involved in nitrogen cycling in rhizospheres and soils, e.g. *Aminobacter* [98], *Rhodococcus* [99], and *Bradyrhizobium* [100]. The latter was also found to be a generalist in soils, exhibiting high abundance in soil profiles analysed at the surface of a similar site located in Nahuelbuta [62]. This together with the observed connectivity of the arid subsurface which experiences 100 times less mean annual precipitation (Pan de Azúcar 12 mm, Nahuelbuta 1469 mm^46^) implies that the humid subsurface between 19 and 34 m depth is likely influenced by the input of microorganisms from surface environments.

Along with the input from the surface along fractures, groundwater can also be an important factor influencing igneous subsurface ecology [17]. For the arid site, located in Pan de Azúcar, groundwater activity is very unlikely. No groundwater was observed during drilling, which reached a maximum depth of 93.5 m. Considering that the deepest subsurface horizon analysed at the arid site was located at 55 m depth, groundwater influence on microbial community composition at the point of sampling can be excluded. However, the lower abundance of genes related to xeric stress in the metagenome of the 26 m deep arid subsurface could imply buffered conditions in the deeper arid subsurface, potentially due to the retention of moisture from rare rain events or temporary groundwater flows within the rock pore space. In the humid site of Nahuelbuta, groundwater could have an impact on the microbial community as water was observed in the borehole at a depth of ~7–9 m. Groundwater tables can vary according to seasonal changes; however, considering the depth of the microbial communities below 19 m and the mean annual rainfall in Nahuelbuta of 1469 mm,^46^ it can be assumed that groundwater is present in all here analysed humid subsurface environments. It is difficult to find evidence on the impact of groundwater on microbial community composition, since there is a large overlap between common groundwater and deep biosphere taxa [18, 101, 102]. In fact, a lot of deep biosphere studies are based on the analysis of groundwater of different depths [14, 18, 19, 22]. Even though the dominance of *Proteobacteria* is a common feature of groundwater environments [101], the high presence of this phylum in the groundwater-unaffected arid deep subsurface makes it impossible to distinguish between shallow groundwater and deep biosphere influences in the humid subsurface.

Chemolithoautotrophy can acquire energy under the absence of photosynthetically produced organic matter. Therefore, we evaluated all the subsurface samples for the presence of chemolithoautotrophs and investigated whether the arid and humid subsurface ecosystems are impacted by surface processes. Furthermore, to analyse which mineral or gas compounds are used for their metabolism, the metagenomic datasets were screened for genes related to the oxidation of inorganic compounds, which serve as an alternative to the degradation of organic matter. Both deep arid subsurface environments, at 26 and 55 m depths, featured potentially chemolithoautotrophic organisms. At 26 m depth, one *Sulfuricurvum* ASV was found, representing the only well-described chemolithoautotroph of the 40 most abundant ASVs. *Sulfuricurvum* is a genus within the phylum *Campylobacterota* and has been reported to oxidize hydrogen and different sulphur compounds [103]. These bacteria have appeared in various subsurface settings, not depending on photosynthetically generated organic matter, including deep biosphere groundwaters of crystalline bedrock and high CO_2_ freshwater aquifers [18, 104]. The metagenomic analysis confirmed the presence of sulphur and hydrogen-oxidizing genes in the 26 m deep subsurface. Several other chemolithoautotrophs related to *Thiobacillus*, *Sulfuriferula*, *Hydrogenophaga*, and the unclassified class 4-29-1 of *Nitrospirota* were recovered from the 55 m deep arid subsurface. *Thiobacillus* is a versatile genus that can oxidize sulphur under aerobic conditions, as well as in anaerobic conditions using nitrate as an electron acceptor [105, 106]. *Sulfuriferula* are also sulphur oxidizers [107]. Certain species of these two genera can have chemolithoautotrophic and heterotrophic metabolisms [108–110]. *Hydrogenophaga* is another previously described group of facultative chemolithoautotrophic bacteria which is known to colonize mineral surfaces and has the metabolic potential to oxidize hydrogen [111, 112]. *Nitrospirota* can use nitrite and hydrogen as electron donors [113]; some have even been proposed to gain energy from the disproportionation of inorganic sulphur [114]. Overall, the presence of hydrogen and sulphur oxidizing taxa and genes in the arid subsurface is in line with previous findings in deeper subsurface rocks [13, 82] and could indicate adaptation to nutrient-limited conditions occurring already at 26 m depth below the desert.

In contrast to the arid subsurface, the taxonomic assessment of the humid subsurface revealed only limited evidence for chemolithoautotrophic organisms. However, genes associated with chemolithoautotrophic growth appeared across all three metagenomes. Carbon fixation genes were abundant across three metagenomes. While the abundance of key genes for the reverse tricarboxylic acid cycle was very low, genes associated with the Calvin cycle and the 3-hydroxypropionate bicycle were present in both arid and humid subsurface horizons. However, the key genes *accABCD*, used in this study to assess the 3-HP bicycle, are not exclusive to this pathway but are also involved in fatty acid synthesis. As a result, while the genetic potential for the 3-hydroxypropionate bicycle is evident, its abundance may be overestimated. Our data suggest genetic potential for sulphur, hydrogen, and carbon monoxide oxidation in the humid and arid subsurface. Sulphur cycling is an important process in deep biosphere environments where reduced sulphur components can be derived from minerals (e.g. FeS, FeS_2_), gases (e.g. H_2_S), and biogenic reduction of sulphate [6]. Studies on deep crystalline aquifers attributed the presence of sulphur-oxidizers to the process of sulphate reduction in groundwater [18]. With pyrite (FeS_2_) being largely absent in the lithology and groundwater likely being absent in the 55 m deep subsurface, the sources for reduced sulphur are unclear. However, sulphate was a soluble anion detected in the arid subsurface and decreased with depth. Therefore, despite the absence of sulphate reducers in our dataset, inorganic reduced sulphur species could become available from biogenic sulphate reduction and fuel chemolithoautotrophic organisms in the arid subsurface. Carbon monoxide oxidation is reported to be an important mechanism in deep fracture waters [14]. Carbon monoxide can be generated from volcanic activity or the biogenic combustion of organic matter. Thus, in line with our metagenomic data, carbon monoxide-based trace gas metabolism can be expected to be present in humid and arid subsurface environments, either fuelled by surface organic matter or geogenic gases.

Despite the lack of organic matter derived from the surface, crystalline deep subsurface environments have often been reported to be enriched in heterotrophic microorganisms, probably due to heterotrophic growth may be overwhelming the much slower metabolisms of chemolithoautotrophic organisms [31, 115]. This was also observed in the arid subsurface in this study, where chemolithoautotrophic taxa were generally less abundant than their heterotrophic counterparts. Chemolithoautotrophic growth fuelled by gas and rock-derived components (e.g. various reduced sulphur compounds, nitrite, ammonium, ferrous iron, and hydrogen) is an important trait of deep biosphere communities in subsurface habitats with little to no surface-derived organic input [19]. This process supplies energy for non-photosynthetic carbon fixation, thereby supporting heterotrophic growth in deep biosphere environments [6, 13]. The presence of chemolithoautotrophs within the non-rare taxa in the 26 and 55 m deep arid subsurface, together with the absence of chemolithoautotrophic organisms in the 19–31.5 m deep humid subsurface, may indicate that oligotrophic conditions and the shift to communities influenced by chemolithoautotrophy appear already at shallower depths underneath arid landscapes. Likely, this is linked indirectly to climatic conditions. In the subsurface of deserts, the limited water and organic matter supply from the surface may lead to increasing isolation. Thus, climate and surface conditions may actively shape microbial community composition in the deeper subsurface, at least within the upper few hundreds of metres of the deep biosphere.

Chemolithoautotrophy is significant for life to inhabit more isolated environments; it can also potentially influence critical weathering processes by directly utilizing minerals or altering the fluid-rock chemistry in the subsurface. Potential nutrient sources in granitic rock could be iron from the mineral biotite. However, chemolithoautotrophs found in the arid subsurface were mostly sulphur, hydrogen, and possibly carbon monoxide oxidizers, while no indication for the direct utilization of iron from redox reactions was found. Since pyrite was not detected in the rock material, we assume potential inorganic nutrients are derived from gases (e.g. H_2_S, H_2_, CO) or biogenically reduced sulphur compounds. These findings are consistent with limited evidence for active mineral dissolution by microbial-driven redox reactions from earlier studies [28]. They may suggest only a minor effect on microbial weathering through such reactions in natural habitats.

However, heterotrophic organisms can also contribute to rock weathering by synthesizing organic and inorganic acids and forming siderophores with chelating abilities. Heterotrophic taxa previously linked to weathering activity were present in arid and humid subsurface horizons. The generalistic ASVs related to *Pseudomonas*, *Burkholderia-Caballeronia-Paraburkholderia*, and *Janthinobacterium* are known to drive mineral dissolution of granite and biotite in different environments, including soils and rock habitats [56, 69, 116]. Metagenomic sequencing supported this by identifying genes related to acidification and chelation across the 1.5 and 26 m deep arid and the 31 m deep humid subsurface. Microbial acidolysis is the most effective and widespread weathering mechanism in soils [56]. Both rock cores are showing alteration in the form of iron oxides on the fracture surfaces; however, due to the extensive hydrothermal overprint in the arid site and the high chemical weathering rates due to water infiltration in the humid site, it is not possible to distinguish to which extent these secondary minerals are formed through microbiological activity.

Our data suggest that microbial weathering potential extends deeper into the subsurface of the critical zone. By utilizing surface-derived organic matter, we propose that heterotrophic organisms possibly contribute to weathering in the humid subsurface. In contrast, in the deeper subsurface of the arid environment, these processes may be fuelled by chemolithoautotrophs. While microbial weathering processes have extensively been studied in various soils [28], rock-associated communities in the subsurface have only been poorly investigated for their weathering potential. This calls for further research investigating the weathering capability of these communities, as genes and processes may differ from the well-characterized mechanisms in soils. Enzymatic assays in our study (see Supplementary Material S2.2, Supplementary Fig. S5) showed that subsurface communities can be active in both arid and humid subsurface ecosystems, suggesting that microbes in these locations can influence deep weathering processes within the critical zone.

## Conclusions

This study assessed and compared the role of deep subsurface microbial communities located in two different climatic settings, providing novel insights into subsurface ecosystem dynamics and into how desert ecosystems transition into deep subsurface environments.

The decline of desert-associated taxa and xeric stress genes in the deeper desert subsurface (26 and 55 m) indicates a depth-related shift towards a subsurface community composition resembling that of the humid subsurface horizons below 19 m depth. However, taxonomic differences between arid and humid deep subsurface remain, with the deeper arid subsurface showing the presence of potentially chemolithoautotrophic organisms. The presence of these taxa may indicate the onset of a shift towards oligotrophic conditions and a limited supply of organic matter within the first 55 m of the arid subsurface, enabling the utilization of inorganic electron donors for carbon fixation from CO_2_. This stands in contrast to the humid subsurface, which likely still receives organic matter from the surface. Together, these patterns indicate that surface climate can indirectly influence deep microbial communities across the first tens to hundreds of metres by creating steeper nutrient gradients with depth under arid conditions.

Metagenomic analysis further emphasized sulphur, hydrogen, and carbon monoxide as the most probable alternative electron donors for subsurface communities in both climates. We also identified the potential for primarily heterotrophic weathering processes in subsurface settings, with acidolysis and chelation being the dominant mechanisms. The fact that deep microbial weathering could be active either under arid conditions, potentially driven by chemolithoautotrophs, or under humid conditions, potentially fuelled by surface-derived organic matter, may indicate a global extent of deep microbial weathering processes and, thus, emphasize its impact within the critical zone.

Together, these findings advance our understanding of global deep subsurface environments in nutrient-depleted igneous systems and their role in critical zone processes, which underlines the importance of investigating the microbial weathering potential of subsurface communities for a complete assessment of deep weathering mechanisms.

## Supplementary Material

Supplementary_material_Horstmann_ycaf199

Table_S15_ycaf199

## Data Availability

The raw 16S rRNA amplicon data and metagenomic data are available at the European Nucleotide Archive (ENA) under the accession number PRJEB76461 or https://www.ebi.ac.uk/ena/browser/view/PRJEB76461. All other displayed data, including curated ASV tables, can be viewed in the supplementary material.
