# Supplementary material for: Microbial ecology of subsurface granitic bedrock: a humid–arid site comparison in Chile
Source: ISME Commun. 2025 Nov 4;5(1):ycaf199. doi: 10.1093/ismeco/ycaf199 (PMC12663961; doi:10.1093/ismeco/ycaf199)
Supplement: Supplementary_material_Horstmann_ycaf199 [file supplementary_material_horstmann_ycaf199.pdf]

Supplementary material

S1. Supplementary methods

S1.1 Drilling procedure

The wireline diamond drilling of both cores was conducted using a standard industry truck-mounted PQ3-sized (85 mm core diameter, 123 mm hole diameter) rotary drilling rig (Sondajes Araos E.I.R.L.). A Long Year Series 4 diamond drilling crown suitable for abrasive rock and a Long Year Series 9 crown for moderately abrasive rock was used. The final actual vertical well depth reached 93.5 m for Pan de Azúcar and 40.8 m for Nahuelbuta, using a standard wireline continuous coring system recovering up to 1.5 m long core runs contained in stainless steel liners. Potable water was used as drilling fluid. The only additive was a suspension of fluorescent particles (RADGLO AFN-09, Radiant Color NV, Houthalen, Belgium) as contamination tracer. This tracer was established by Friese et al. (2017) and allows the quantification of potential contaminations during drilling operations by comparing particle counts of drilling fluid and core samples. After recovery from the well, each core run (~ 1.5 m) was separated into 20–30 cm long samples using an angle grinder, hammer, and chisel under sterile conditions. Samples were immediately stored in vacuum-sealed bags under frozen (-20 °C) conditions.

S 1.2 Ion chromatography (IC), electric conductivity (EC) and pH

Cation and anion analysis was performed on a SYKAM Compact IC system (Sykam Chromatographie, Fürstenfeldbruck, Germany) with a detection limit of 0.1 mg l^{-1}. Ions were leached using a protocol previously applied on desert soils[2] with samples dried at 50 °C for 12 h and sieved to <2 mm grain size. The leaching was performed in a 1:5 ratio of 5 g rock powder to 25 mL Milli-Q water, incubating the samples for 1.5 h on an orbital shaker before ion chromatographic measurement. Standards for the detection of cations (Roth, Multi-Element IC Standard Solution, covering lithium, sodium, ammonium, potassium, magnesium, and calcium)

and anions (Sykam, covering fluoride, chloride, nitrite, bromide, nitrate, phosphate, and sulfate) were used. The samples were run in triplicates to estimate the error based on the standard deviation.

The same leachates as from the ion analysis were used for pH measurement using a Toledo FEP20-Basic FiveEasyTM Plus pH Meter with the pH electrode LE407, as well as for electric conductivity measurement using the WTW 2FD460 Multi 3420 Multiparameter Meter with the WTW TetraCon® 925 IDS Conductivity Probe.

S1.3 Enzymatic activity

Enzymatic activity across microbial communities in rock samples was assessed using a fluorescein diacetate (FDA) hydrolytic activity assay based on Green et al. (2006). 1 g of rock powder was mixed with 12.5 ml of 60 mM sodium phosphate buffer (Na_3PO_4, pH 7.6) and 0.25 ml of 4.9 mM FDA solution (20 mg FDA in 10 ml acetone). The samples were mixed and placed in an incubator for 3 h at 37°C. After incubation, 1 ml of acetone was added to the mixture to terminate FDA hydrolysis. The mixture was then centrifuged at 8,820 *g* for 5 min and filtered through a PP filter. Through absorbance measurement at a wavelength of 490 nm using a spectrophotometer, enzyme activity was quantified using a standard curve with concentrations ranging from 0.2 to 20 µg ml^{-1} through absorbance measurement at a wavelength of 490 nm using a spectrophotometer.

Blanks were measured using rock powder of the corresponding sample with phosphate buffer, only phosphate buffer with FDA solution, and only phosphate buffer without FDA solution to detect potential background signals. Mean values of released fluorescein from all rock and solution blanks were then subtracted from actual sample values.

S2. Supplementary results

S2.1 Soluble ions, electric conductivity, and pH

Water soluble ion concentrations were generally higher for the arid than the humid samples (Fig. S3). Sodium and potassium ions showed a relatively constant appearance with depth for both sites, with sodium concentrations of 0.56-0.78 mM in the arid subsurface and 0.08-0.12 mM in the humid subsurface, and potassium concentrations of 0.01-0.13 mM in the arid subsurface and 0.02-0.01 mM in the humid subsurface. Divalent calcium and magnesium cations were only detectable in the three arid samples of 12 m, 25 m, and 55 m depth. While the 12 m and 25 m samples showed similar values of 0.11-0.13 mM for calcium and around 0.01 mM for magnesium, the most profound sample showed higher concentrations of 0.19 mM and 0.02 mM for calcium and magnesium, respectively. Chloride and sulfate were the most abundant soluble anions. Chloride concentrations in the humid subsurface were relatively constant and ranged between 0.01 and 0.02 mM, while in arid one, values were up to two orders of magnitude higher and decreased with depth from 0.53 mM at 1.5 m to 0.19 mM at 55 m. Sulfate concentrations behaved similarly, with constantly low concentrations in the humid subsurface ranging from 0.002 mM to 0.004 mM, while they are significantly higher in the arid subsurface decreasing from 0.04 mM at 1.5 m to 0.01 mM at 55 m. Nitrate could only be measured in humid samples and was generally very low, with concentrations of 0.003 at 19 m and 0.002 at 31 m depth. Nitrate levels at 31.5 m depth were around 0.001, just below the detection limit (0.1 mg l^{-1} = 0.0016 mM NO_3^-), while no value was obtained for the 34 m deep sample.

Obtained pH values from the same leachates that were used for ion chromatography were generally basic, ranging from 7.6 to 9.4. The lowest pH of 7.6 was observed for the shallow arid sample at 1.5 m depth. The deeper arid samples had a higher pH of around 9.4, while the pH of the humid samples ranged from 7.9 to 8.6 (Fig. S4). Electric conductivity in samples from both sites was constant, did not change with depth, and was generally low, ranging between

8.5-14.1 μS cm^{-1} for the humid subsurface and 10 times higher values of 93.3-130.2 μS cm^{-1} in the arid subsurface.

S2.2 Microbial activity

Microbial activity was measured using the fluorescein diacetate hydrolytic activity (FDA) assay. For 6 of 8 samples, microbial activity was measurable, no reliable values were obtained for the 1.5 m and 12 m deep arid samples. Activity for the deeper arid samples was measurable, releasing 1.32 μg fluorescein g^{-1} h^{-1} at 26 m depth and 0.41 μg fluorescein g^{-1} h^{-1} at 55 m depth (Fig. S5). In the humid subsurface, activity partially increased with depth with 0.63 μg of fluorescein released g^{-1} h^{-1} at 19 m depth, 1.62 μg of fluorescein released g^{-1} h^{-1} at 31 m depth and the highest value of 3.44 μg of fluorescein released g^{-1} h^{-1} at 31.5 m depth (Fig. S5). In 34 m depth, activity decreases to 1.10 μg of fluorescein released g^{-1} h^{-1}.

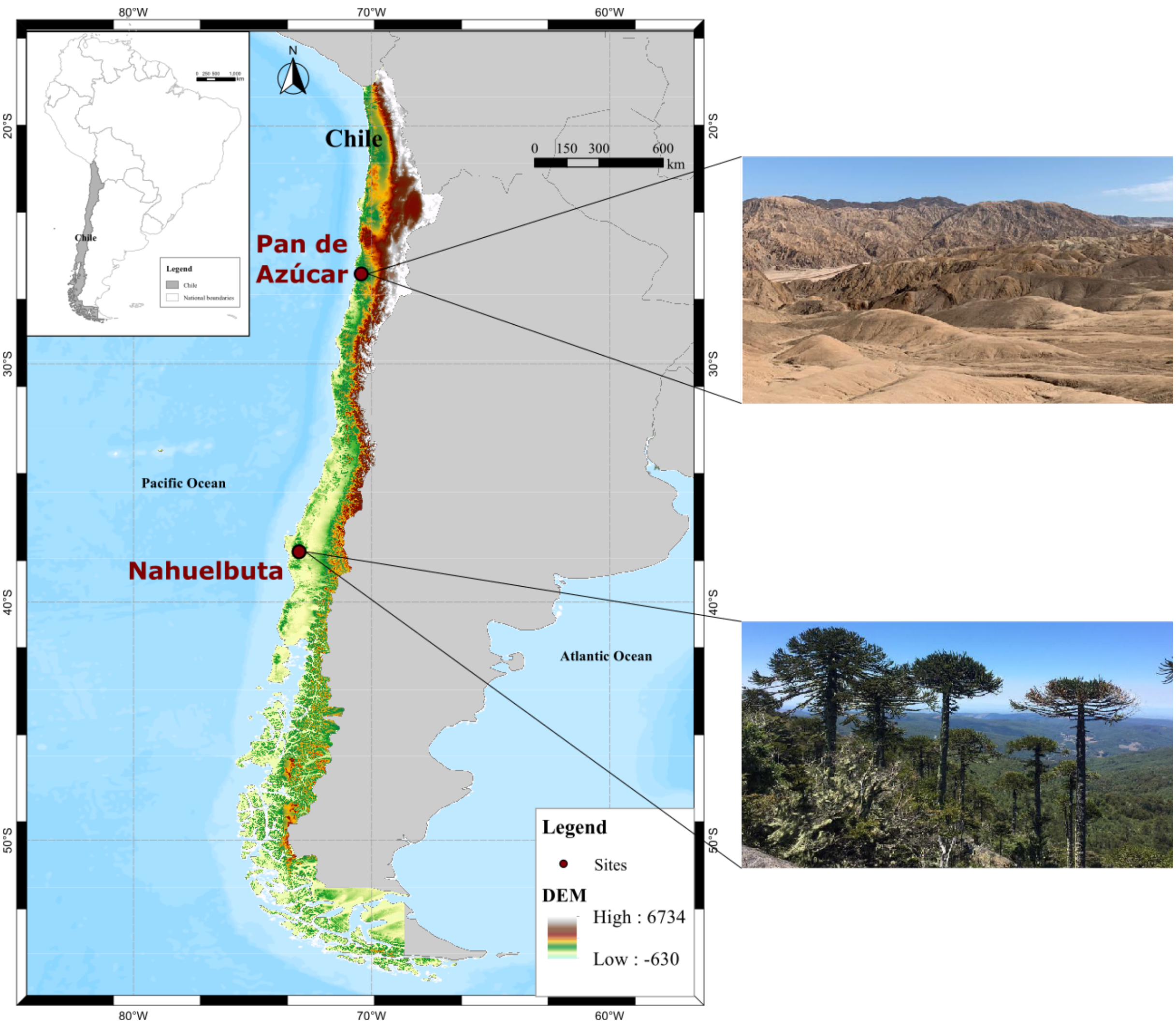

Figure S1 Map modified from Wang et al. (2024) showing the locations of the drilling with an overview picture of both sites. Pan de Azúcar in the north represents arid conditions while Nahuelbuta in the south represents humid conditions.

**KEGG Orthology (K0)**	**Name**	**Function**
K18008	hydA	hydrogen oxidation
K00437	hydB	hydrogen oxidation
K03605	hyaD, hybD	hydrogen oxidation
K03620	hyaC	hydrogen oxidation
K17999	hydC	hydrogen oxidation
K00534	E1.12.7.2S	hydrogen oxidation
K06281	hyaB, hybC	hydrogen oxidation
K17230	fccA	sulfur oxidation
K17229	fccB	sulfur oxidation
K17218	sqr	sulfur oxidation
K17222	soxA	sulfur oxidation
K17223	soxX	sulfur oxidation
K17226	soxY	sulfur oxidation
K17227	soxZ	sulfur oxidation
K17224	soxB	sulfur oxidation
K05301	sorA	sulfur oxidation
K00386	sorB	sulfur oxidation
K21307	soeA	sulfur oxidation
K21308	soeB	sulfur oxidation
K21309	soeC	sulfur oxidation
K24299	tcdH	sulfur oxidation
K20760	scnA	sulfur oxidation
K20761	scnB	sulfur oxidation
K20762	scnC	sulfur oxidation
K20150	cyc2	iron oxidation
K03518	coxS	carbon monoxide oxidation
K03519	coxM	carbon monoxide oxidation
K09386	coxG	carbon monoxide oxidation
K10944	amoA	ammonia oxidation
K11180	dsrA	sulfate reduction
K11181	dsrB	sulfate reduction
K23077	dsrC	sulfate reduction
K00394	aprA	sulfate reduction
K00395	aprB	sulfate reduction
K00367	narB	nitrate reduction
K00370	narG, narZ	nitrate reduction
K00371	narH, narY	nitrate reduction
K02567	napA	nitrate reduction
K02568	napB	nitrate reduction
K03385	nrfA	nitrite reduction
K04015	nrfD	nitrite reduction
K00362	nirB	nitrite reduction
K00363	nirD	nitrite reduction
K00368	nirK	nitrite reduction
K04561	norB	nitric oxide reduction
K02305	norC	nitric oxide reduction
K00376	nosZ	nitrous oxide reduction
K02274	coxA, ctaD	oxygen reduction

K02274,K15408	coxA, ctaD	oxygen reduction
K02274,K02298,K15408	coxA, ctaD	oxygen reduction
K15408	coxAC	oxygen reduction
K00399	mcrA	methanogenesis
K02588	nifH	nitrogen fixation
K00855	PRK, prkB	Calvin cycle
K00855,K00876	PRK, prkB	Calvin cycle
K01602	rbcS	Calvin cycle
K01601	rbcL	Calvin cycle
K01601,K08965	rbcL	Calvin cycle
K15232	ccsA	rTCA
K00194	cdhD, acsD	WL pathway
K01961	accC	3-HP bicycle
K01961,K01968,K11263	accC	3-HP bicycle
K01961,K01965	accC	3-HP bicycle
K01962	accA	3-HP bicycle
K01962,K01963	accA, accD	3-HP bicycle
K01963	accD	3-HP bicycle
K02160	accB, bccP	3-HP bicycle
K01571,K02160	accB, bccP	3-HP bicycle
K00627,K02160,K07402	accB, bccP	3-HP bicycle
K00627,K00645,K01572,K02160	accB, bccP	3-HP bicycle
K01958,K02160,K17490	accB, bccP	3-HP bicycle
K14534	abfD	3HP/4HB cycle

Table S1 KEGG Orthology(KO) numbers of key genes that were selected for analysed across samples and their association with specific pathways.

Kegg Orthology (K0)	Name	Sub-function	Function
K03673	dsbA	Acidification	Acidification
K06136	pqqB	Acidification	Acidification
K06139	pqqE	Acidification	Acidification
K00117	gcd	Acidification	Acidification
K01647	gltA	Acidification	Acidification
K01910	citC	Acidification	Acidification
K07539	oah	Acidification	Acidification
K00034	gdh	Acidification	Acidification
K06151	gad alpha	Acidification	Acidification
K06152	gad beta	Acidification	Acidification
K07345	fimA	Acidification	Acidification
K15540	ecpD	Acidification	Acidification
K02664	pilO	Acidification	Acidification
K00845	glk	Acidification	Acidification
K25031	gntK	Acidification	Acidification
K07399	resB	Acidification	Acidification
K10531	pvdA	Chelation	Chelation
K12241	pchG	Chelation	Chelation
K12242	pchC	Chelation	Chelation
K00625	pta	Chelation	Chelation
K00925	ackA	Chelation	Chelation
K00156	poxB	Chelation	Chelation
K05847	opuA	Solute transport	Compatible solute accumulation
K05846	opuBD	Solute transport	Compatible solute accumulation
K05845	opuC	Solute transport	Compatible solute accumulation
K05020	opuD	Solute transport	Compatible solute accumulation
K02000	proV	Solute transport	Compatible solute accumulation
K02001	proW	Solute transport	Compatible solute accumulation
K02002	proX	Solute transport	Compatible solute accumulation
K02819	TreB	Solute transport	Compatible solute accumulation
K00697	otsA	Solute synthesis	Compatible solute accumulation
K01087	otsB	Solute synthesis	Compatible solute accumulation
K06718	ectA	Solute synthesis	Compatible solute accumulation

K00836	ectB	Solute synthesis	Compatible solute accumulation
K06720	ectC	Solute synthesis	Compatible solute accumulation
K04078	groES	Chaperone synthesis	Stress response
K04077	groEL	Chaperone synthesis	Stress response
K04043	dnaK	Chaperone synthesis	Stress response
K03686	dnaJ	Chaperone synthesis	Stress response
K04047	dsp	Oxidative stress protection	Stress response
K04565	SOD1	Oxidative stress protection	Stress response
K04564	SOD2	Oxidative stress protection	Stress response
K03781	katE	Oxidative stress protection	Stress response
K03782	katG	Oxidative stress protection	Stress response
K13631	soxS	Oxidative stress protection	Stress response
K13487	wspA	Stress sensoring/regulation	Stress response
K03087	rpoS	Stress sensoring/regulation	Stress response
K03088	rpoE	Stress sensoring/regulation	Stress response
K13798	rpoB	Stress sensoring/regulation	Stress response
K06149	uspA	Stress sensoring/regulation	Stress response
K01991	wza	Biofilm formation	Biofilm formation
K16692	wzc	Biofilm formation	Biofilm formation
K16554	exoP	Biofilm formation	Biofilm formation
K07699	spo0A	Sporulation	Sporulation
K06376	spo0E	Sporulation	Sporulation
K06413	spoVK	Sporulation	Sporulation
K06386	spoIIQ	Sporulation	Sporulation

Table S2 KEGG Orthology numbers for key genes related to microbial weathering(according to Uroz et al., 2022), xeric adaption, biofilm formation and sporulation.

**Climate**	**Site**	**Sample ID**	**Depth**	**Particle concentrations**		**Potential contamination [µl g^{-1}]**
				**drilling fluid [l^{-1}]**	**core material [g^{-1}]**	
Arid	Pan de Azúcar	AZ 3	1.58-1.66 m	$8.3 * 10^{10}$	$3.6 * 10^{5}$	4.31
		AZ 11	11.80-11.90 m	$4.0 * 10^{11}$	$9.0 * 10^{4}$	0.22
		AZ 24	25.80-25.96 m	$9.3 * 10^{10}$	$1.6 * 10^{5}$	1.80
		AZ 46	54.70-54.80 m	$1.2 * 10^{12}$	$8.5 * 10^{4}$	0.07
Humid	Nahuelbuta	NA 29	19.17-19.25 m	$2.3 * 10^{11}$	$3.3 * 10^{5}$	1.40
		NA 39.0	30.30-30.48 m	$2.8 * 10^{11}$	$8.9 * 10^{5}$	3.15
		NA 39.18	30.48-30.78 m	$2.8 * 10^{11}$	$4.2 * 10^{5}$	1.48
		NA 41	33.66-33.72 m	$7.0 * 10^{10}$	$1.0 * 10^{5}$	1.48

Table S3 Amount of liner fluid that potentially entered the inner section of each core run. Contamination is expressed in the volume of liner fluid per gram of rock powder.

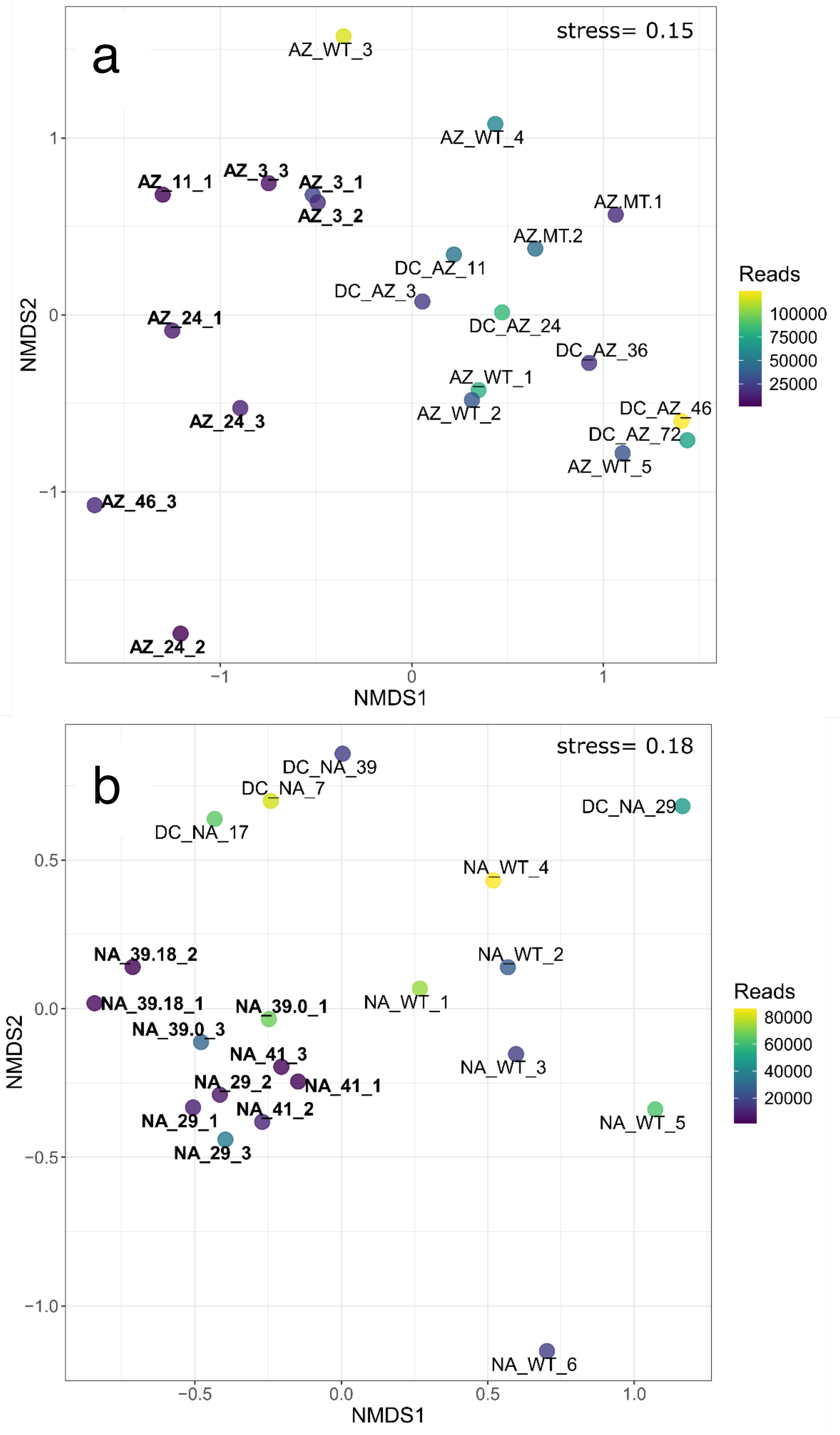

Figure S2 NMDS plot showing the similarity between controls (water tank = WT & drill control = DC) and in situ communities for each site (a= arid, b= humid). Plots are based on a Bray Curtis dissimilarity matrix calculated with subsampled read counts. Distances are displayed in a two-dimensional space.

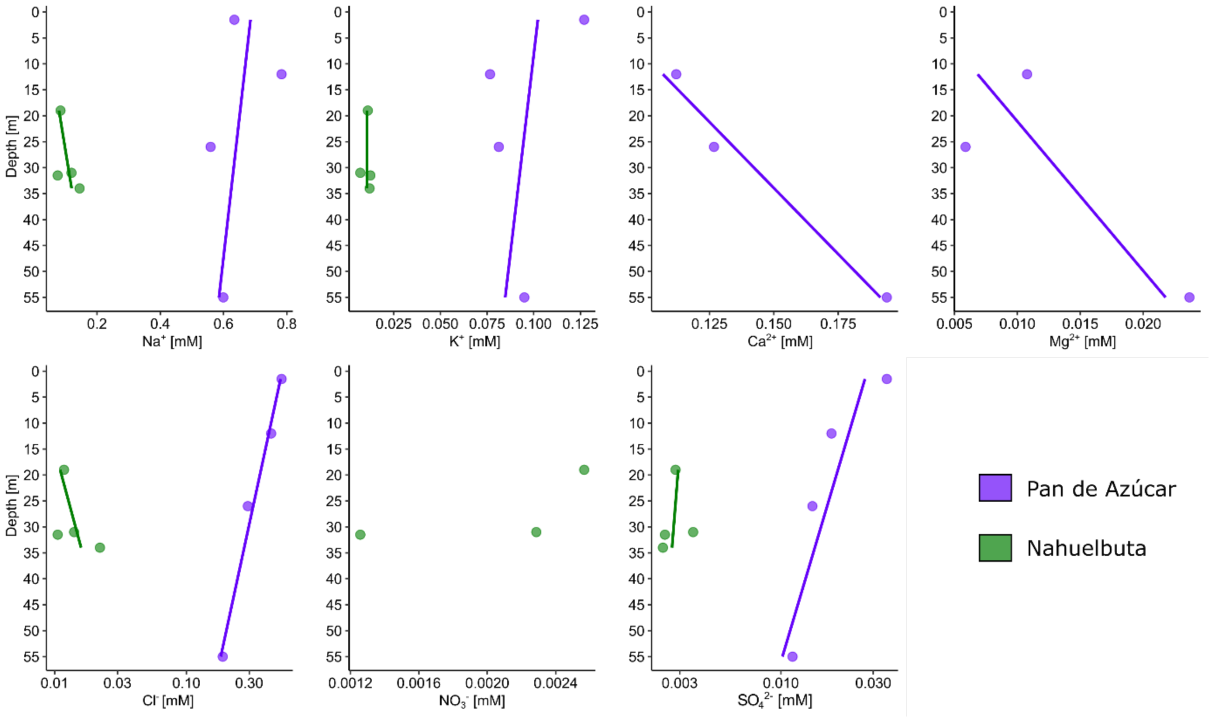

Figure S3 Cation and anion concentrations of all core samples from Pan de Azúcar and Nahuelbuta. Ions were leached using Milli-Q water on rock powder. Note the logarithmic scale on the x-axis for the chloride and sulfate concentrations.

Climate	Site	Sample	Depth [m]	Replicate	Cl [mM]	NO3 [mM]	SO4 [mM]	Na [mM]	K [mM]	Mg [mM]	Ca [mM]
Humid	Nahuelbuta	NA_29	19	1	0.012	0.003	0.003	0.078	0.011		
		NA_29	19	2	0.012	0.003	0.003	0.086	0.011		
		NA_29	19	3	0.012	0.003	0.003	0.090	0.011		
		NA_39.0	31	1	0.014	0.002	0.003	0.113	0.007		
		NA_39.0	31	2	0.014	0.002	0.004	0.120	0.007		
		NA_39.0	31	3	0.014	0.002	0.004	0.125	0.007		
		NA_39.18	31.5	1	0.011	0.001	0.003	0.075	0.013		
		NA_39.18	31.5	2	0.011	0.001	0.003	0.076	0.012		
		NA_39.18	31.5	3	0.011	0.001	0.002	0.077	0.012		
		NA_41	34	1	0.022		0.002	0.143	0.013		
		NA_41	34	2	0.022		0.003	0.146	0.012		
		NA_41	34	3	0.022		0.002	0.145	0.011		
Arid	Pan de Azucar	AZ_3	1.5	1	0.529		0.035	0.665	0.128		
		AZ_3	1.5	2	0.527		0.035	0.620	0.124		
		AZ_3	1.5	3	0.528		0.035	0.618	0.129		
		AZ_11	12	1	0.443		0.018	0.788	0.077	0.011	0.110
		AZ_11	12	2	0.440		0.018	0.791	0.082	0.010	0.114
		AZ_11	12	3	0.438		0.018	0.772	0.071	0.011	0.112
		AZ_24	26	1	0.293		0.015	0.558	0.084	0.006	0.123
		AZ_24	26	2	0.293		0.015	0.570	0.082	0.006	0.125
		AZ_24	26	3	0.293		0.014	0.549	0.078	0.006	0.132
		AZ_46	55	1	0.189		0.012	0.596	0.094	0.025	0.196
		AZ_46	55	2	0.189		0.012	0.598	0.093	0.022	0.188
		AZ_46	55	3	0.189		0.011	0.604	0.098	0.024	0.197

Table S4 Values of cation and anion concentrations from Fig. S3.

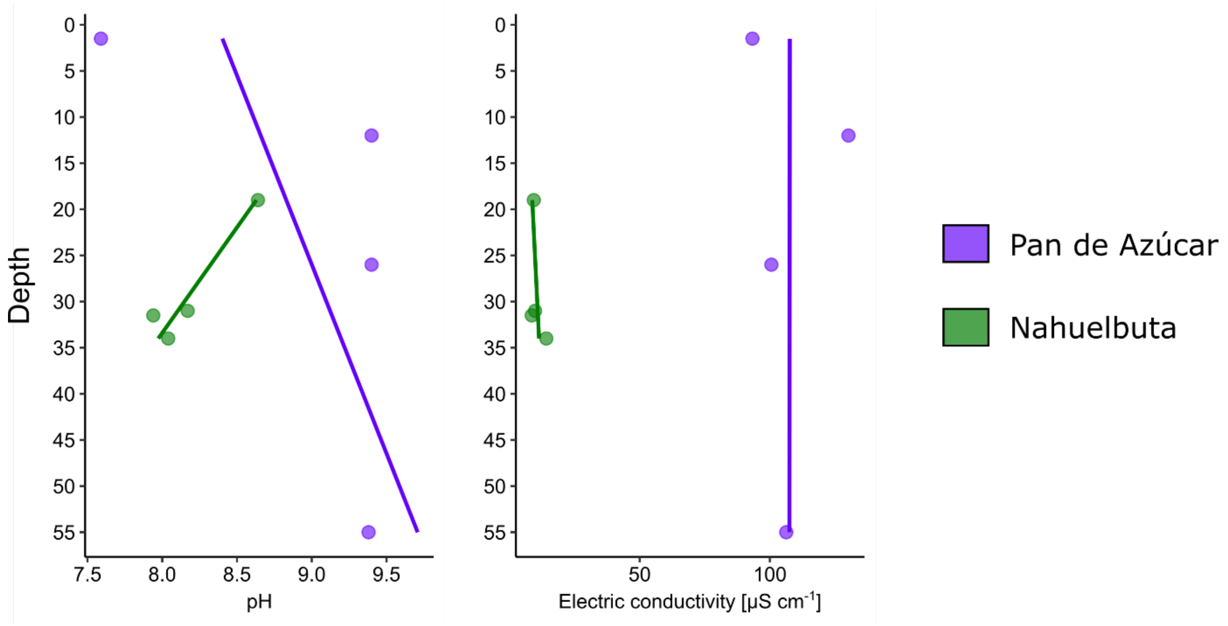

Figure S4 pH and electric conductivity measured on the water leachates from the ion measurements.

Climate	Site	Sample	Depth [m]	pH	EC
Humid	Nahuelbuta	NA_29	19	8.64	9.3
		NA_39.0	31	8.17	9.8
		NA_39.18	31.5	7.94	8.5
		NA_41	34	8.04	14.1
Arid	Pan de Azucar	AZ_11	12	9.4	130.2
		AZ_24	26	9.4	100.6
		AZ_3	1.5	7.59	93.3
		AZ_46	55	9.38	106.3

Table S5 Values of pH and electric conductivity from Fig. S4.

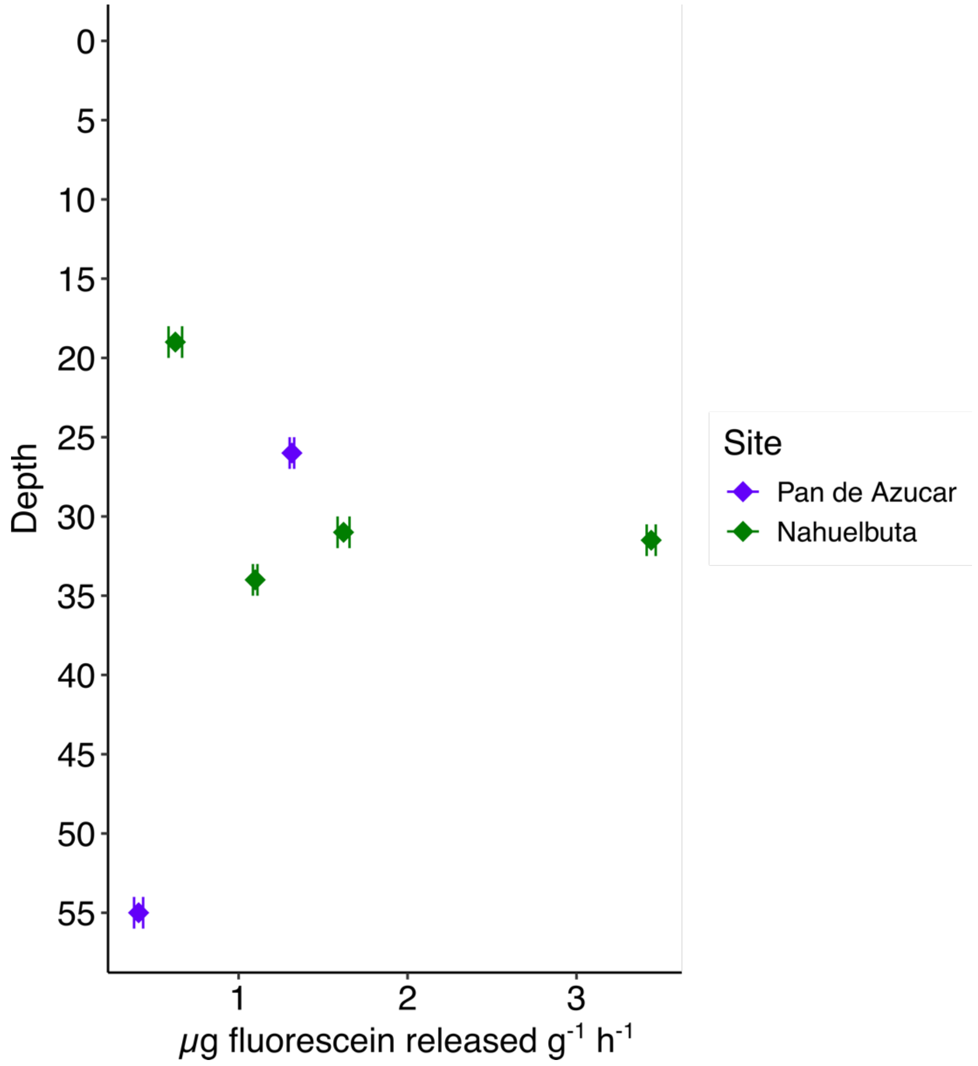

Figure S5 Microbial activity measured using fluorescein diacetate hydrolytic activity (FDA). Values below the detection limit (AZ 3, AZ 11 and NA 41) are not displayed.

Climate	Site	Sample	Depth [m]	µg fluorescein released g^{-1} h^{-1}	sd
Arid	Pan de Azucar	PdA-3	1.5		
		PdA-11	7		
		PdA-24	26	1.32	0.01
		PdA-46	55	0.41	0.03
Humid	Nahuelbuta	NA-29	19	0.63	0.04
		NA-39-0	31	1.62	0.04
		NA-39-18	31.5	3.44	0.03
		NA-41	34	1.10	0.01

Table S6 Values for activity measured using fluorescein diacetate hydrolytic activity (FDA) from Fig. S5.

Climate	Site	Sample	Depth [m]	Observed	Shannon	gene copies g^{-1} rock	sd
Arid	Pan de Azucar	AZ_11	12	35	2.75		
		AZ_24	26	105	3.98		
		AZ_3	1.5	279	4.86	466893.31	54807.01
		AZ_46	55	103	4.21		
Humid	Nahuelbuta	NA_29	19	172	4.39	15246.28	4499.36
		NA_39.0	31	185	4.37	66424.53	9013.94
		NA_39.18	31.5	63	3.07		
		NA_41	34	131	4.13		

Table S7 Alpha diversity (observed and Shannon) and microbial abundance (qPCR) data.

Kingdom	Phylum	relative abundance
Bacteria	Proteobacteria	50.39%
Bacteria	Actinobacteriota	25.59%
Bacteria	Firmicutes	8.12%
Bacteria	Bacteroidota	4.91%
Bacteria	Chloroflexi	1.69%
Archaea	Crenarchaeota	1.40%
Bacteria	Verrucomicrobiota	1.37%
Bacteria	Planctomycetota	1.02%
Bacteria	Cyanobacteria	0.98%
Bacteria	Acidobacteriota	0.94%
Bacteria	Gemmatimonadota	0.78%
Bacteria	Campilobacterota	0.54%
Bacteria	Patescibacteria	0.50%
Bacteria	Myxococcota	0.31%
Archaea	Thermoplasmatota	0.24%
Bacteria	Methylomirabilota	0.24%
Bacteria	Bdellovibrionota	0.23%
Bacteria	Nitrospirota	0.20%
Archaea	Euryarchaeota	0.14%
Other		< 0.1%

Table S8 Overall phylum abundance across samples.

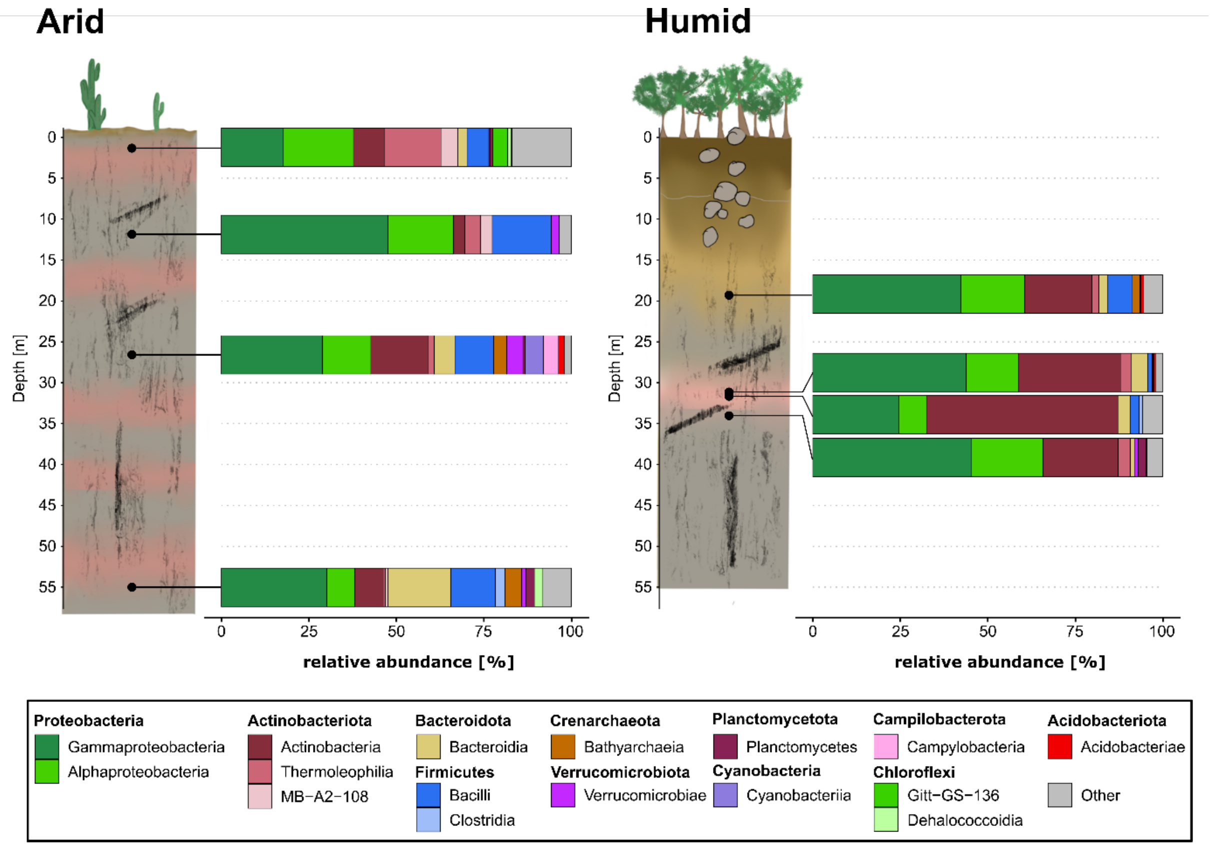

Figure S6 Class level microbial composition of arid and humid subsurface samples and their location within the profile indicated on a schematic illustration of the rock cores.

ASV	NA_29	NA_39.0	NA_39.18	NA_41	AZ_3	AZ_11	AZ_24	AZ_46
ASV_10	0.79	6.93	8.00	2.60	0.11	0.85	10.60	5.93
ASV_100	0.03	4.29	0.00	0.00	0.00	0.00	0.00	0.00
ASV_126	0.00	2.47	10.40	0.00	0.00	0.00	0.58	0.00
ASV_1315	0.00	0.00	0.00	0.00	0.00	11.32	0.00	0.00
ASV_187	3.16	0.00	0.00	0.00	0.04	0.00	0.90	0.00
ASV_19	0.01	0.10	1.53	3.55	0.03	0.00	0.00	0.00
ASV_207	2.09	0.10	0.00	2.83	0.01	0.00	0.00	0.00
ASV_21	0.32	0.08	5.53	0.00	0.08	1.69	0.66	0.01
ASV_23	0.02	0.00	2.76	0.07	0.00	0.00	4.08	0.00
ASV_2376	0.00	0.00	0.00	0.00	0.00	4.31	0.00	0.00
ASV_24	0.00	0.00	3.71	0.36	0.00	0.00	0.00	0.00
ASV_26	3.05	0.80	0.00	0.65	0.00	0.00	0.00	0.00
ASV_260	0.00	0.00	0.00	0.00	0.00	0.00	5.08	0.00
ASV_276	0.00	0.00	0.00	0.00	0.00	10.08	0.00	0.00
ASV_323	0.00	0.00	0.00	0.00	0.00	0.00	4.30	0.00
ASV_356	0.00	0.00	0.00	0.00	0.00	0.00	1.35	3.22
ASV_369	0.00	0.00	0.00	0.00	0.00	0.00	0.00	4.99
ASV_39	0.25	1.85	2.29	0.09	1.24	0.00	1.93	0.00
ASV_4	0.00	0.01	0.00	0.00	7.72	19.78	4.21	0.02
ASV_412	0.00	0.16	0.00	0.00	0.00	4.85	0.00	0.00

**ASV_451**	0.00	0.00	0.00	0.00	0.00	0.00	0.00	4.16
**ASV_5**	0.01	0.00	0.00	0.26	3.78	2.93	0.00	0.48
**ASV_50**	3.65	2.11	1.00	0.51	1.05	0.00	0.00	0.00
**ASV_52**	4.43	5.48	0.00	10.94	0.00	0.00	0.58	0.00
**ASV_582**	0.00	0.00	1.45	0.00	0.00	14.70	0.00	0.00
**ASV_59**	1.69	0.81	0.11	0.53	1.14	0.00	0.24	0.00
**ASV_596**	0.00	0.00	0.00	0.00	0.00	4.31	0.00	0.00
**ASV_66**	3.85	0.12	4.97	1.74	0.00	0.15	2.94	2.62
**ASV_7**	0.37	0.01	0.74	0.60	0.00	4.39	0.62	0.01
**ASV_70**	4.69	2.01	0.00	1.47	0.00	0.00	0.00	0.00
**ASV_73**	4.07	2.56	25.80	0.00	0.00	0.00	0.00	0.00
**ASV_76**	1.28	4.15	0.00	1.63	0.00	0.00	0.00	0.00
**ASV_77**	1.34	1.16	0.00	2.36	0.00	0.00	0.00	0.28
**ASV_87**	0.15	2.66	1.13	3.91	0.00	0.00	0.00	0.00
**ASV_9**	0.00	0.00	0.00	0.00	0.11	0.00	3.29	2.86
**ASV_91**	3.72	0.75	0.00	0.48	0.01	0.00	0.66	0.00
**ASV_92**	0.00	4.39	0.00	0.00	0.00	0.00	0.00	0.19
**ASV_93**	0.52	1.59	0.34	5.66	0.10	0.00	0.00	0.00
**ASV_95**	1.77	1.05	0.00	1.94	0.67	0.00	0.00	0.00
**ASV_96**	1.24	4.11	0.00	0.00	0.00	0.00	0.00	0.00

Table S9 Relative abundance [%] of most abundant ASVs displayed in Fig. 3.

**ASV**	**Kingdom**	**Phylum**	**Class**	**Order**	**Family**	**Genus**
**ASV_10**	Bacteria	Actinobacteriota	Actinobacteria	Micrococcales	Micrococcaceae	Pseudarthrobacter
**ASV_100**	Bacteria	Proteobacteria	Gammaproteobacteria	Pseudomonadales	Moraxellaceae	Acinetobacter
**ASV_126**	Bacteria	Actinobacteriota	Actinobacteria	Micrococcales	Micrococcaceae	Paenarthrobacter
**ASV_1315**	Bacteria	Proteobacteria	Gammaproteobacteria	Oceanospirillales	Halomonadaceae	Halomonas
**ASV_187**	Bacteria	Actinobacteriota	Actinobacteria	Micrococcales	Micrococcaceae	Arthrobacter
**ASV_19**	Bacteria	Proteobacteria	Alphaproteobacteria	Rhizobiales	Beijerinckiaceae	Methylobacterium-Methylorubrum
**ASV_207**	Bacteria	Proteobacteria	Gammaproteobacteria	Burkholderiales	Comamonadaceae	
**ASV_21**	Bacteria	Proteobacteria	Gammaproteobacteria	Pseudomonadales	Moraxellaceae	Enhydrobacter
**ASV_23**	Bacteria	Proteobacteria	Gammaproteobacteria	Burkholderiales	Burkholderiaceae	Burkholderia-Caballeronia-Paraburkholderia
**ASV_2376**	Bacteria	Actinobacteriota	Thermoleophilia	Solirubrobacterales	67-14	
**ASV_24**	Bacteria	Proteobacteria	Gammaproteobacteria	Enterobacterales		
**ASV_26**	Bacteria	Proteobacteria	Alphaproteobacteria	Sphingomonadales	Sphingomonadaceae	Sphingomonas
**ASV_260**	Bacteria	Cyanobacteria	Cyanobacteriia			
**ASV_276**	Bacteria	Proteobacteria	Alphaproteobacteria	Sphingomonadales	Sphingomonadaceae	Sphingobium
**ASV_323**	Bacteria	Campilobacterota	Campylobacteria	Campylobacterales	Sulfurimonadaceae	Sulfuricurvum
**ASV_356**	Bacteria	Firmicutes	Bacilli	Bacillales	Planococcaceae	Planomicrobium

**ASV_369**	Bacteria	Bacteroidota	Bacteroidia	Flavobacteriales	Flavobacteriaceae	Flavobacterium
**ASV_39**	Bacteria	Proteobacteria	Alphaproteobacteria	Sphingomonadales	Sphingomonadaceae	Sphingopyxis
**ASV_4**	Bacteria	Proteobacteria	Gammaproteobacteria	Pseudomonadales	Pseudomonadaceae	Pseudomonas
**ASV_412**	Bacteria	Proteobacteria	Gammaproteobacteria	Pseudomonadales	Pseudomonadaceae	Pseudomonas
**ASV_451**	Bacteria	Bacteroidota	Bacteroidia	Flavobacteriales	Flavobacteriaceae	Flavobacterium
**ASV_5**	Bacteria	Proteobacteria	Gammaproteobacteria	Pseudomonadales	Pseudomonadaceae	Pseudomonas
**ASV_50**	Bacteria	Actinobacteriota	Actinobacteria	Corynebacteriales	Nocardiaceae	Rhodococcus
**ASV_52**	Bacteria	Proteobacteria	Gammaproteobacteria	Pseudomonadales	Pseudomonadaceae	Pseudomonas
**ASV_582**	Bacteria	Firmicutes	Bacilli	Bacillales	Bacillaceae	Bacillus
**ASV_59**	Bacteria	Actinobacteriota	Actinobacteria	Corynebacteriales	Nocardiaceae	Rhodococcus
**ASV_596**	Bacteria	Proteobacteria	Alphaproteobacteria	Caulobacterales	Caulobacteraceae	
**ASV_66**	Bacteria	Proteobacteria	Gammaproteobacteria	Burkholderiales	Oxalobacteraceae	Janthinobacterium
**ASV_7**	Bacteria	Proteobacteria	Gammaproteobacteria	Burkholderiales	Burkholderiaceae	Burkholderia-Caballeronia-Paraburkholderia
**ASV_70**	Bacteria	Proteobacteria	Alphaproteobacteria	Rhizobiales	Xanthobacteraceae	Afipia
**ASV_73**	Bacteria	Actinobacteriota	Actinobacteria	Micrococcales	Micrococcaceae	
**ASV_76**	Bacteria	Proteobacteria	Gammaproteobacteria	Pseudomonadales	Pseudomonadaceae	Pseudomonas
**ASV_77**	Bacteria	Proteobacteria	Alphaproteobacteria	Rhizobiales	Xanthobacteraceae	Bradyrhizobium
**ASV_87**	Bacteria	Actinobacteriota	Actinobacteria	Corynebacteriales	Nocardiaceae	Gordonia
**ASV_9**	Bacteria	Firmicutes	Bacilli	Lactobacillales	Listeriaceae	Listeria
**ASV_91**	Bacteria	Proteobacteria	Alphaproteobacteria	Rhizobiales	Rhizobiaceae	Aminobacter
**ASV_92**	Bacteria	Proteobacteria	Gammaproteobacteria	Pseudomonadales	Moraxellaceae	Acinetobacter
**ASV_93**	Bacteria	Proteobacteria	Alphaproteobacteria	Rhizobiales	Xanthobacteraceae	Bradyrhizobium
**ASV_95**	Bacteria	Proteobacteria	Gammaproteobacteria	Burkholderiales	Comamonadaceae	Variovorax
**ASV_96**	Bacteria	Proteobacteria	Gammaproteobacteria	Burkholderiales	Oxalobacteraceae	Herbaspirillum

Table S10 Complete taxonomy of most abundant ASVs displayed in Fig. 3.

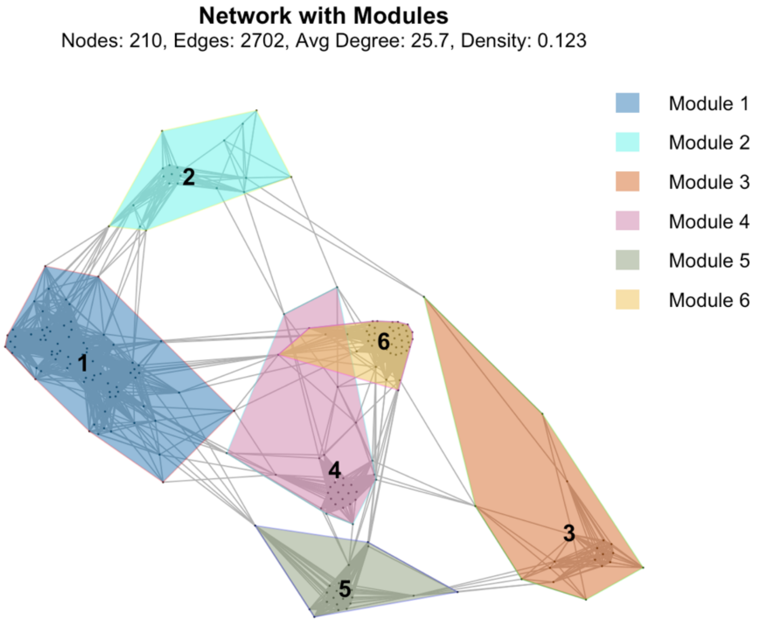

Figure S7 Visualization of network and statistics used for Fig. 4 indicating the 6 different modules in coloured polygons.

Function	NA39	AZ3	AZ24
sulfur oxidation	417	296	654
hydrogen oxidation	89	27	5
carbon monoxide oxidation	359	867	508
ammonia oxidation	0	3	1
oxygen reduction	779	1344	664
sulfate reduction	6	13	0
nitrate reduction	679	190	228
nitrite reduction	560	527	291
nitric oxide reduction	93	13	58
nitrous oxide reduction	106	22	7
nitrogen fixation	38	1	1
Calvin cycle	53	20	140
rTCA	1	46	1
3-HP bicycle	707	628	453

Table S11 Relative abundance of functions based on read counts found for selected genes (see Table S1). For better visualization. read counts were normalized to reads per million (rpm).

Function	NA 39	AZ 3	AZ 24
iron_aquisition-iron_transport	89	102	33
iron_aquisition-heme_transport	13	8	4
iron_aquisition-heme_oxygenase	1	0	0
iron_aquisition-siderophore_synthesis	22	11	9
iron_aquisition-siderophore_transport	189	84	47
iron_aquisition-siderophore_transport_potential	69	33	22
iron_gene_regulation	314	389	64
iron_oxidation	1	1	0
possible_iron_oxidation_and_possible_iron_reduction	0	0	0
probable_iron_reduction	0	0	0
iron_reduction	0	0	0
iron_storage	45	66	7
magnetosome_formation	0	0	0

Table S12 FeGenie output showing normalized gene counts for iron related functions.

Gene	humid 31 m	arid 1.5 m	arid 26 m	Sub-function	Funciton
exoP	82.31	51.54	18.28	Biofilm formation	Biofilm formation
wza	113.48	24.58	135.38	Biofilm formation	Biofilm formation
wzc	28.98	28.07	9.57	Biofilm formation	Biofilm formation
ectA	13.05	13.09	4.62	Solute synthesis	Compatible solute accumulation
ectB	124.53	32.16	13.44	Solute synthesis	Compatible solute accumulation
ectC	15.93	19.80	1.51	Solute synthesis	Compatible solute accumulation
otsA	105.42	416.91	19.79	Solute synthesis	Compatible solute accumulation
otsB	148.39	188.69	119.36	Solute synthesis	Compatible solute accumulation
opuA	201.04	247.77	89.68	Solute transport	Compatible solute accumulation
opuBD	190.67	256.61	43.55	Solute transport	Compatible solute accumulation
opuC	148.51	240.20	39.25	Solute transport	Compatible solute accumulation
opuD	158.26	31.15	38.50	Solute transport	Compatible solute accumulation
proV	144.39	54.40	185.70	Solute transport	Compatible solute accumulation
proW	96.37	44.27	146.35	Solute transport	Compatible solute accumulation
proX	311.27	42.46	162.26	Solute transport	Compatible solute accumulation
treB	0	0	0	Solute transport	Compatible solute accumulation
citC	0	0	0	Acidification	Microbial weathering
dsbA	42.97	6.91	83.66	Acidification	Microbial weathering
ecpD	40.72	0	0	Acidification	Microbial weathering
fimA	63.45	0	0	Acidification	Microbial weathering

gad alpha	117.29	13.27	166.13	Acidification	Microbial weathering
gad beta	66.70	11.17	157.85	Acidification	Microbial weathering
gcd	124.72	69.24	5.16	Acidification	Microbial weathering
gdh	47.59	151.71	2.37	Acidification	Microbial weathering
glk	224.33	316.52	177.21	Acidification	Microbial weathering
gltA	569.88	682.25	810.02	Acidification	Microbial weathering
gntK	0	0	0	Acidification	Microbial weathering
oah	0	0	0	Acidification	Microbial weathering
pilO	36.78	54.23	71.83	Acidification	Microbial weathering
pqqB	49.65	19.94	1.61	Acidification	Microbial weathering
pqqE	104.67	41.97	10.54	Acidification	Microbial weathering
resB	273.67	151.37	290.65	Acidification	Microbial weathering
ackA	146.14	178.99	140.43	Chelation	Microbial weathering
pchC	0	0	0	Chelation	Microbial weathering
pchG	0	0	0	Chelation	Microbial weathering
poxB	88.31	191.07	154.09	Chelation	Microbial weathering
pta	0	0	0	Chelation	Microbial weathering
pvdA	93.18	4.16	1.29	Chelation	Microbial weathering
spo0A	0	0	0	Sporulation	Sporulation
spo0E	0	0	0	Sporulation	Sporulation
spoIIQ	0	0	0	Sporulation	Sporulation
spoVK	0	0	0	Sporulation	Sporulation
dnaJ	289.84	472.81	188.93	Chaperone synthesis	Stress response
dnaK	725.08	802.81	555.71	Chaperone synthesis	Stress response
groEL	919.87	1001.77	710.12	Chaperone synthesis	Stress response
groES	119.72	178.08	93.23	Chaperone synthesis	Stress response
dps	89.68	55.52	163.44	Oxidative stress protection	Stress response

katE	595.80	140.79	890.99	Oxidative stress protection	Stress response
katG	541.84	194.35	367.43	Oxidative stress protection	Stress response
SOD1	48.59	46.65	58.50	Oxidative stress protection	Stress response
SOD2	153.63	217.46	132.80	Oxidative stress protection	Stress response
soxS	0	0	0	Oxidative stress protection	Stress response
rpoB	0	11.42	0	Stress sensoring/regulation	Stress response
rpoE	2119.66	4802.96	1827.24	Stress sensoring/regulation	Stress response
rpoS	49.84	9.22	97.31	Stress sensoring/regulation	Stress response
uspA	20.48	5.17	0.97	Stress sensoring/regulation	Stress response
wspA	18.30	0.14	0	Stress sensoring/regulation	Stress response

Table S13 Abundance of genes related to microbial weathering, xeric adaptation, biofilm formation and sporulation (in rpm) across all metagenomic samples. Microbial weathering genes were selected according to Uroz et al. (2022).

Raw file name	Sample	Raw read pairs	Deduplicated read pairs	QC read pairs	Total contig length [bp]	% of assembled reads
mgshot-S8613Nr1	humid 31m	49705080	21893639	19738303	74152949	34.5
mgshot-S8613Nr2	arid 1.5m	70260492	37076708	34759452	128308498	34.3
mgshot-S8613Nr3	arid 26m	62029060	16122050	13339333	13904152	30.3
				**total**	216365599	
				**total genes**	229247	

Table S14 Summary of the shotgun metagenomic sequencing statistics.
